# A chromatin remodelling SWI/SNF subunit, Snr1, regulates neural stem cell determination and differentiation

**DOI:** 10.1242/dev.201484

**Published:** 2023-06-30

**Authors:** Sophie E. Keegan, Julie Haskins, Andrew J. Simmonds, Sarah C. Hughes

**Affiliations:** ^1^ Department of Cell Biology, Faculty of Medicine and Dentistry, University of Alberta, Edmonton, AB T6G 2H7, Canada; ^2^ Department of Medical Genetics, Faculty of Medicine and Dentistry, University of Alberta, Edmonton, AB T6G 2H7, Canada

**Keywords:** *Drosophila*, SMARCB1, SWI/SNF complex, Snr1, Neural stem cell, Neuroepithelial cells, Neuroblast, Differentiation, Optic lobe

## Abstract

Coordinated spatio-temporal regulation of the determination and differentiation of neural stem cells is essential for brain development. Failure to integrate multiple factors leads to defective brain structures or tumour formation. Previous studies suggest changes of chromatin state are needed to direct neural stem cell differentiation, but the mechanisms are unclear. Analysis of Snr1, the *Drosophila* orthologue of SMARCB1, an ATP-dependent chromatin remodelling protein, identified a key role in regulating the transition of neuroepithelial cells into neural stem cells and subsequent differentiation of neural stem cells into the cells needed to build the brain. Loss of Snr1 in neuroepithelial cells leads to premature neural stem cell formation. Additionally, loss of Snr1 in neural stem cells results in inappropriate perdurance of neural stem cells into adulthood. Snr1 reduction in neuroepithelial or neural stem cells leads to the differential expression of target genes. We find that Snr1 is associated with the actively transcribed chromatin region of these target genes. Thus, Snr1 likely regulates the chromatin state in neuroepithelial cells and maintains chromatin state in neural stem cells for proper brain development.

## Teaser

**Summary:** The chromatin remodelling SWI/SNF subunit Snr1 acts as a regulator of expression of transcription factors that direct cell differentiation in the developing brain.

## INTRODUCTION

**Fig. 1.**
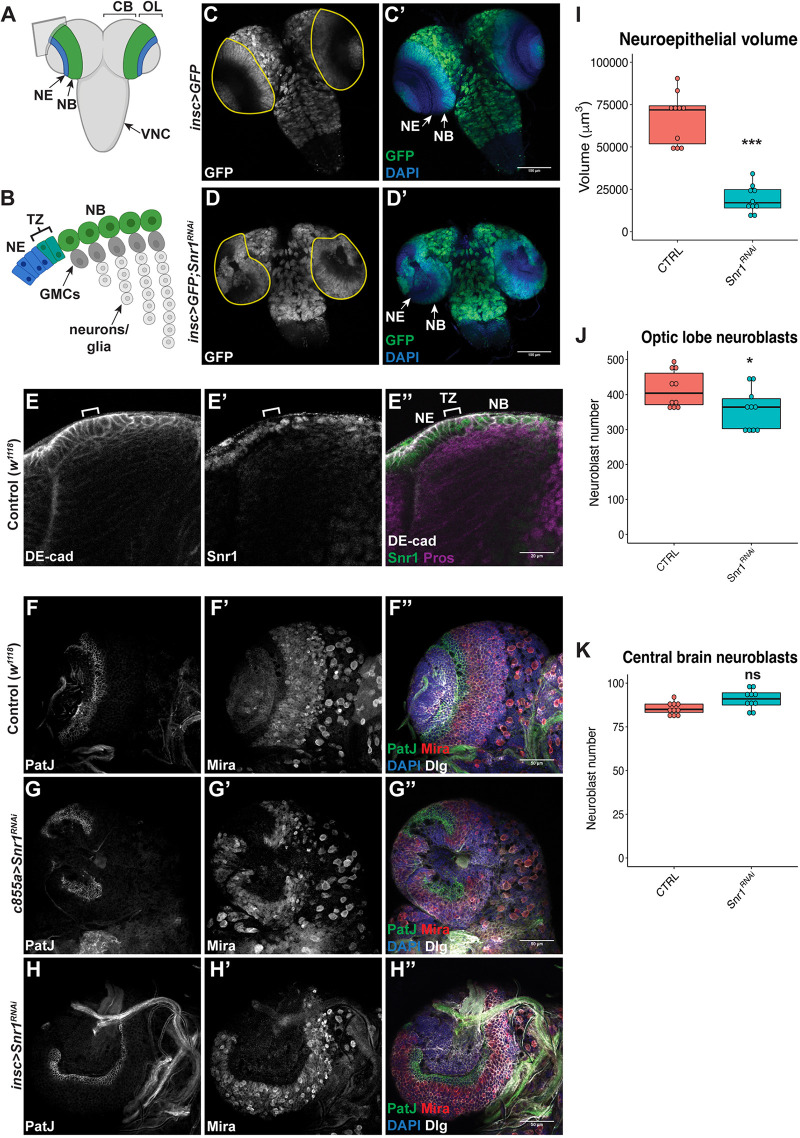
**Snr1 is expressed in neuroepithelial and neuroblast cells in the optic lobe, and is required for optic lobe development.** (A) Diagram of the *Drosophila* third instar larval brain. Optic lobe neuroepithelial cells (NE) represented in blue and medulla neuroblasts (NBs) in green. CB, central brain; OL, optic lobe; VNC, ventral nerve cord. Gray square indicates the plane of cross-section shown in B. Created with BioRender.com. (B) Diagram of a cross-section of the optic lobe showing the transition zone (TZ), ganglion mother cells (GMCs) and neurons located in the medulla. Created with BioRender.com. (C,C′) Neuroblasts expressing GFP (green). (D,D′) GFP (green) and *Snr1*^RNAi^ are expressed in all neuroblasts. The OL is outlined in yellow in C,D. Scale bars: 100 µm in C-D′. (E-E″) Larval optic lobe imaged in cross-section showing the transition zone (bracket) from neuroepithelial cells to neuroblasts. The transition zone is identified by a change in cell shape, as marked by DE-cadherin (DE-cad). Scale bar: 20 µm. (F-F″) Wild type *w^1118^* brain. (G-G″) Snr1 knocked down in neuroepithelial cells (c855a) by *Snr1*^RNAi^. (H-H″) *Snr1* knocked down in neuroblasts (insc) by *Snr1*^RNAi^. (F-H″) Neuroepithelial cells marked by PatJ (green), neuroblasts marked by Miranda (Mira) (red). In F″, G″ and H″, DAPI is in blue and Discs Large (Dlg) is in white. Scale bars: 50 µm. (I) Neuroepithelial volume in control (66,759±15,201 µm^3^) and c855a>*Snr1*^RNAi^ (19,138±8075 µm^3^) brains. *P*=1e-5, *n*=10. (J) Optic lobe neuroblast numbers in control (416±53) and insc>*Snr1*^RNAi^ (358±58) brains. *P*=0.03, *n*=10. (K) Central brain neuroblasts in control (85±4) and insc>*Snr1*^RNAi^ (91±5) brains. *P*=0.07, *n*=10. *P<0.05, ***P<0.001; ns, not significant.

Neural stem cells that form the brain integrate complex signalling inputs to produce the large variety of mature neuronal and glial cell types (Molyneaux et al., 2007). This requires precise spatial and temporal coordination of proliferation and differentiation. Failure to do so leads to altered connections (defects) and over-proliferation (tumours). For example, in the developing murine cerebral cortex, neuroepithelial cells divide symmetrically to increase the size of the stem cell pool. They then convert to radial glial cells (mammalian neural stem cells), which divide asymmetrically and respond to signals driving differentiation and specification of neuron subtypes of the adult brain (Farkas and Huttner, 2008; Gaspard et al., 2008; Hevner, 2006; McConnell, 1995; Ypsilanti et al., 2021). There is analogous patterning of the *Drosophila melanogaster* larval optic lobe, where conserved overlapping signalling pathways specify the generation of each type of neuron (Brand and Livesey, 2011; Doe, 2017; Egger et al., 2010). These neurons contribute to the adult optic lobe receives input from photoreceptors and transmits visual information to the central brain (Nériec and Desplan, 2016). The adult optic lobe is divided into four neuropils that process visual information: the medulla, the lamina, the lobula and the lobula plate (Morante and Desplan, 2004). The medulla, the largest part of the optic lobe, arises from symmetrically dividing neuroepithelial cells during larval development (Egger et al., 2007) (Fig. 1A). In the outer neuroepithelium, proneuronal genes are expressed in a region termed the transition zone (Fig. 1B; bracket) that converts neuroepithelial cells into neural stem cells (Egger et al., 2010; Yasugi et al., 2008). Neural stem cells (neuroblasts in *Drosophila*) divide asymmetrically to produce ganglion mother cells (GMCs), which then divide once to produce the neurons and glia of the optic lobe (Fig. 1B). Neuroblasts in the outer medulla express a series of transcription factors serving as temporal and spatial cues to specify the extensive neuronal diversity in their progeny (Erclik et al., 2017; Li et al., 2013). Although several transcription factors that define the optic lobe have been identified, the mechanisms that coordinate the generation of the vast diversity of cell types is less understood. The accessibility and similarity of the optic lobe to mammalian cortex development provides a powerful model for investigating the regulation of neural stem cell determination and differentiation.

SMARCB1 (SWI/SNF-related, matrix-associated, actin-dependent regulator of chromatin, subfamily B, member 1) is a core subunit of Switch/Sucrose Non-Fermentable (SWI/SNF) complexes, a conserved family of multi-subunit, ATP-dependent chromatin remodelling complexes (Ho and Crabtreee, 2010). SWI/SNF complexes regulate gene expression by promoting DNA accessibility via sliding or ejecting histones. Mammalian SWI/SNF, called Brahma/Brahma-related gene 1-associated factor (BAF) complexes, act in opposition to Polycomb-mediated transcriptional repression to maintain epigenetic stability during developmental stages (Ho and Crabtree, 2010; Kennison and Tamkun, 1988; Tamkun et al., 1992). In addition to an essential role during development, mutations in genes encoding BAF complex subunits are detected in more than 20% of cancers (Ho and Crabtree, 2010; Ho et al., 2009; Kadoch et al., 2013). SMARCB1 is an essential regulator of embryonic development in mice with loss linked to formation of central nervous system tumours (Klochendler-Yeivin et al., 2000; Roberts et al., 2000). Although SMARCB1 is a core subunit of the BAF complex, the BAF complex maintains residual chromatin remodelling activity even when SMARCB1 is lost (Gatchalian et al., 2018; Mashtalir et al., 2018; Michel et al., 2018). Residual complex binding at some enhancers alters accessibility at bivalent promoters, resulting in an increase in expression of genes promoting proliferation at the expense of differentiation (Nakayama et al., 2017; Wang et al., 2017). In *Drosophila*, the ortholog of SMARCB1, *Snf5-related 1* (*Snr1*), is also essential for maintaining gene expression during development (Dingwall et al., 1995; Marenda et al., 2003; Zraly et al., 2003). Previous studies have shown a role for Snr1 in the differentiation of type II neuroblasts and proliferation in wing discs (Eroglu et al., 2014; Koe et al., 2014; Xie et al., 2017).

We found that Snr1 is required for optic lobe development, regulating both neuroepithelial cells and neuroblasts. Reduced Snr1 levels caused neuroepithelial cells to prematurely differentiate into neuroblasts, which subsequently failed to differentiate at the appropriate stage of brain development. We identified multiple neuronal progenitor specific transcription factors with reduced expression due to decreased Snr1 levels. Notably, we found that Snr1 is associated with the genetic loci of these specific transcription factors along with an active histone mark. These results suggest that Snr1 acts as a regulator of expression of transcription factors that direct cell differentiation in the developing brain.

## RESULTS

### Snr1 regulates optic lobe development

*Snr1* null mutants are larval lethal (Dingwall et al., 1995). Thus, we determined the effect of reduced Snr1 levels in neuroblasts, by targeting *Snr1* with RNA interference (RNAi) during *Drosophila* larval development. The GAL4-UAS system (Brand and Perrimon, 1993) was used to express *Snr1*^RNAi^ in all neuroblasts using *inscuteable*-GAL4 (*insc*-GAL4), which reduced *Snr1* mRNA levels by 77% (Fig. S1B,C). Cells expressing *insc*-GAL4 were co-labelled with green fluorescent protein (GFP), showing their location in the optic lobe, central brain and ventral nerve cord (Fig. 1C,C′). *Snr1*^RNAi^ expression resulted in misshapen optic lobes, while other structures were similar to control (Fig. 1D,D′ versus Fig. 1C,C′). Additional GAL4 lines with expression in the optic lobe were tested and had similar disruption of optic lobe development (see Fig. 1). As this suggested Snr1 plays a role in optic lobe development, this region of the brain was analysed further. We found Snr1 expression in neuroepithelial cells and neuroblasts on the surface of the brain (Fig. 1E′), suggesting a role for Snr1 in neural progenitors.

As Snr1 was expressed in neuroepithelial cells and neuroblasts, we focused on cell type-specific *Snr1*^RNAi^ in neuroepithelial cells (c855a-GAL4) and neuroblasts (*insc*-GAL4). When *Snr1* was knocked down in neuroepithelial cells, the optic lobe was reduced in size and disorganized (Fig. 1G-G″). The outer neuroepithelium, marked by PatJ (PatJ+), was reduced compared with the control (Fig. 1G, I). Conversely, neuroblast *Snr1*^RNAi^ did not affect the neuroepithelium (Fig. 1H), while the region of optic lobe neuroblasts, marked by Miranda (Mira) (Egger et al., 2007), appeared misshapen (Fig. 1H′). The number of optic lobe neuroblasts was slightly reduced when *Snr1*^RNAi^ was expressed in neuroblasts (Fig. 1J). As *insc*-GAL4 is expressed in all neuroblasts, the number of central brain neuroblasts was also measured and did not show a significant change when Snr1 expression was reduced (Fig. 1K). The development of the optic lobe was affected by loss of Snr1 expression in neuroepithelial cells or neuroblasts, demonstrating the role of Snr1 in the two cell types or in regulating the transition between cell states.

### Neuroepithelial to neuroblast transition is disrupted in Snr1-deficient cells

**Fig. 2.**
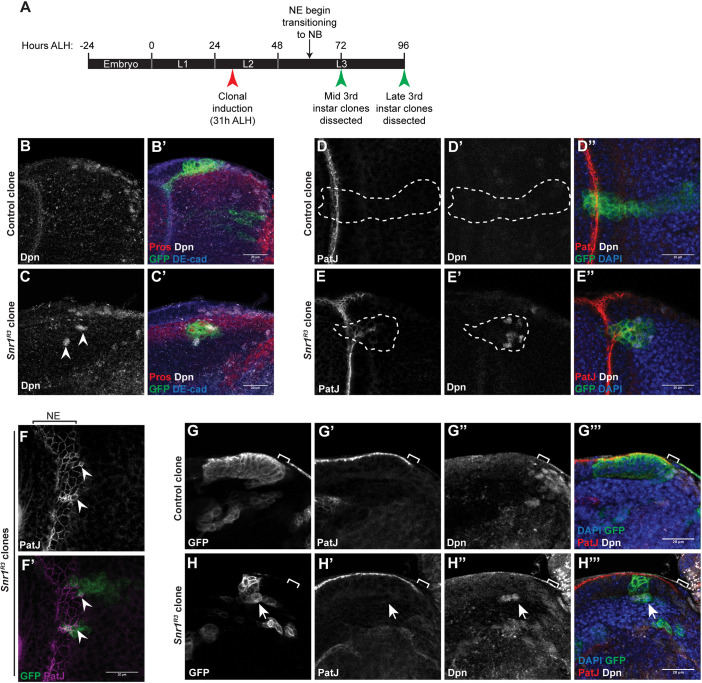
**Snr1 is required for the transition from neuroepithelial cells to neuroblasts.** (A) Timeline of larval development showing timing of clonal induction and dissections after larval hatching (ALH). (B-C′) *Snr1* mutant clones in the optic lobe have altered location and morphology. (B,B′) Control MARCM clone at surface of brain imaged in cross-section. (C,C′) *Snr1*^R3^ MARCM clone located deeper inside the brain showing ectopic expression of Deadpan (Dpn; arrowheads). Cell junctions are marked by DE-cad, neuroblasts are marked by Dpn, MARCM clones are marked by GFP and GMCs are marked by Pros. (D-E″) Snr1 mutant clones deep in the medulla express markers for neuroepithelial cells and neuroblasts. (D-D″) Control optic lobe MARCM clone marked by GFP. Clone is outlined by a white dashed line. (E-E″) *Snr1*^R3^ optic lobe MARCM clone marked by GFP. Clone is outlined by a white dashed line. Neuroepithelial cells marked by PatJ (red) and neuroblasts marked by Dpn (white). (F-F′) *Snr1*^R3^ clone cells on the surface of the brain are smaller (arrowheads) and express neuroepithelial (NE) cell marker. Neuroepithelial cell junctions are marked by PatJ (magenta) and MARCM clones are marked by GFP (green). (G-H‴) MARCM clones imaged in cross-section at mid third instar stage. Brackets indicate transition zone. Arrows indicate ectopic Dpn^+^ cells. Scale bars: 20 µm.

The changes observed during brain development caused by *Snr1*^RNAi^ could be attributed to altered differentiation or proliferation. To differentiate between these possibilities, mosaic analysis with a repressible cell marker (MARCM) (Lee and Luo, 1999) was used to generate clonal populations of *Snr1* null mutant cells (*Snr1^R3^*) marked by membrane-targeted GFP (GFP^+^) to trace the development of Snr1-deficient cells in comparison with control cells in the same tissue. Clones were induced at a time point when neuroepithelial cells make up the majority of the optic lobe and analysed later, after the neuroepithelial to neuroblast transition (Fig. 2A) (Egger et al., 2007). Clones were generated in mitotic cells, close to the surface of the brain where dividing neuroepithelial cells, neuroblasts and GMCs are located (Egger et al., 2010). In control brains, GFP^+^ clones extended from the surface into the medulla (control, Fig. 2B,B′). In contrast, *Snr1^R3^* mutant clones were more rounded and can be disconnected from the surface of the brain (*Snr1^R3^*, Fig. 2C,C′). As the location of *Snr1^R3^* clones deeper inside the brain indicated the neuroepithelial to neuroblast transition was disrupted, clones were marked for neuroepithelial cells (PatJ) and neuroblasts (Deadpan, Dpn) (Bier et al., 1992), and imaged deep inside the medulla, where neurons are typically present, and neuroepithelial cells and neuroblasts are absent (Fig. 2D-D″). Mutant cell clones, deep in the medulla, were found to inappropriately have PatJ^+^ or Dpn^+^ cells (*Snr1^R3^*, Fig. 2E-E″), whereas cells in wild-type clones did not (control, Fig. 2 D-D″). *Snr1^R3^* clones also exhibited altered morphology compared with control clones. On the surface of the optic lobe, neuroepithelial cell junctions (PatJ^+^) outlined cells of uniform size (Fig. 2F). In *Snr1^R3^* clones, cells were smaller and disorganized (Fig. 2F′, arrowhead), suggesting adhesion was affected by loss of Snr1. To determine whether the morphology of *Snr1^R3^* clones was a result of altered timing of the transition to neuroblasts, clones were imaged at an earlier time point (mid third instar versus late third instar) (Fig. 2A,G-H‴). Neuroepithelial cells in the *Snr1^R3^* clones at this stage transitioned prematurely (before the transition zone) to neuroblasts (Fig. 2H″) and were extruded from the neuroepithelium (Fig. 2H). Thus, Snr1 was required to regulate the timing of the transition from neuroepithelial cells to neuroblasts.

### Snr1-deficient neuroblasts failed to differentiate

**Fig. 3.**
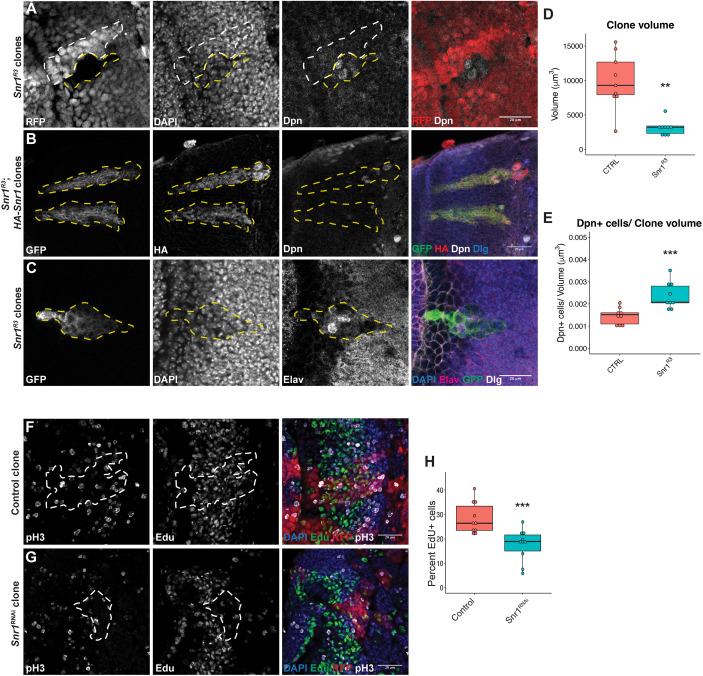
**Snr1 mutant cells maintain expression of neuroblast marker and mis-express neuronal marker.** (A) Twin spot clone labelled with RFP, DNA marked with DAPI and neuroblasts marked with Deadpan (Dpn). *Snr1*^R3^ clones are negative for RFP (outlined with a yellow dashed line) and the corresponding wild-type clones are RFP positive (outlined with a white dashed line). In merged images, RFP is in red and Dpn is in white. (B) Expression of HA-Snr1 partially rescued clone morphology and ectopic Dpn expression in *Snr1*^R3^ clones. MARCM clones express GFP (green). In the merged image, Dpn is shown in white, HA in red and Dlg in blue. (C) *Snr1*^R3^ MARCM clone with disrupted Elav expression (magenta). Scale bars: 20 µm in A-C. (D) Volume of control (9894±4011 µm^3^) and *Snr1*^R3^ (3119±1093 µm^3^) clones. *P*=1e-3, *n*=9. (E) Number of Dpn^+^ cells per volume of control (1.45e-3±3.72e-4) and *Snr1*^R3^ (2.38e-3±5.98e-4) clones. *P*=8e-4, *n*=9. (F) Control FLP-out clone labelled with EdU. (G) FLP-out clone expressing *Snr1*^RNAi^ labelled with EdU. (F,G) Mitotic cells labelled with phospho-histone H3 (pH3). Clones are outlined with white dashed lines. Scale bars: 20 µm in F,G. (H) Percentage of EdU^+^ cells in control (29±6) and *Snr1*^RNAi^ (18±7) clones. *P*=1e-3, *n*=10. **P<0.01, ***P<0.001.

Although loss of Snr1 disrupted the transition from neuroepithelial cells to neuroblasts, the optic lobe was also affected when *Snr1*^RNAi^ was expressed in neuroblasts after this transition occurred, indicating a role for Snr1 in regulating neuroblast cell fates (Fig. 1H-H″). To compare the fate of cells from a common progenitor cell directly, somatic mosaic lineage tracing was used to compare *Snr1^R3^* cells with wild-type cells in ‘twin spot’ clones. Cells in clones homozygous for *Snr1^R3^* null allele (RFP^−^) contained cells expressing the neuroblast marker Dpn, whereas wild-type (RFP^+^) cells did not express Dpn (Fig. 3A). The volume of *Snr1^R3^* clones was reduced compared with control clones (Fig. 3D), whereas the number of Dpn^+^ cells per clone volume in *Snr1^R3^* clones was increased (Fig. 3E). This phenotype was specific to loss of *Snr1*, as expression of HA-tagged *Snr1* in *Snr1^R3^* clones reduced the ectopic Dpn expression in deep brain clones and suppressed aberrant morphology of the clones (Fig. 3B versus Fig. 2D′). Expression of postmitotic neuron marker embryonic lethal abnormal visual (Elav) (Bier et al., 1988; Robinow and White, 1991) was also disrupted in *Snr1^R3^* clones (Fig. 3C). Abnormal perdurance of the neuroblast marker Dpn and disrupted expression of the neuron marker Elav in *Snr1^R3^* clones suggested that these cells failed to differentiate at the appropriate stage during optic lobe development.

As *Snr1^R3^* null mutant clones were smaller than control (Fig. 3A,D), we investigated the effect of reduced Snr1 on proliferation and cell death. In FLP-out clones expressing nuclear RFP and *Snr1*^RNAi^ (Struhl and Basler, 1993), the average number of cells was reduced by 67% compared with control (51.5 versus 163.8 cells per clone, *P*=0.002, Fig. S2C-C″,E). There was no significant change in the percentage of mitotic cells in *Snr1*^RNAi^ clones (Fig. S2C,F). Similarly, no increase in cell death in the *Snr1*^RNAi^ clones that would account for the decreased number of cells was detected (Fig. S2C′,G). We also expressed the P35 inhibitor of apoptosis alone or in *Snr1*^RNAi^ clones (Hay et al., 1994) (Fig. S2B-B″,D-D″). Expression of P35 alone did reduce the percentage of dying cells per clone compared with the control (Fig. S2B′,G), whereas the percentage of dying cells in clones expressing P35 and *Snr1*^RNAi^ simultaneously was unchanged from control clones (Fig. S2D′,G). As neither the proportion of mitotic or dying cells could account for the decreased size of the *Snr1*^RNAi^ clones, 5-ethynyl-2′-deoxyuridine (EdU) labelling was used to measure the proportion of cells undergoing DNA synthesis (Fig. 3F,G). The *Snr1*^RNAi^ clones had a decreased proportion of EdU-positive cells relative to control clones (Fig. 3H). Therefore, the reduced size of *Snr1*^RNAi^ clones was likely due to a reduction in the rate of cells entering the cell cycle, suggesting that neuroblasts are proliferating more slowly despite an increase in neuroblast numbers (Fig. 3E).

**Fig. 4.**
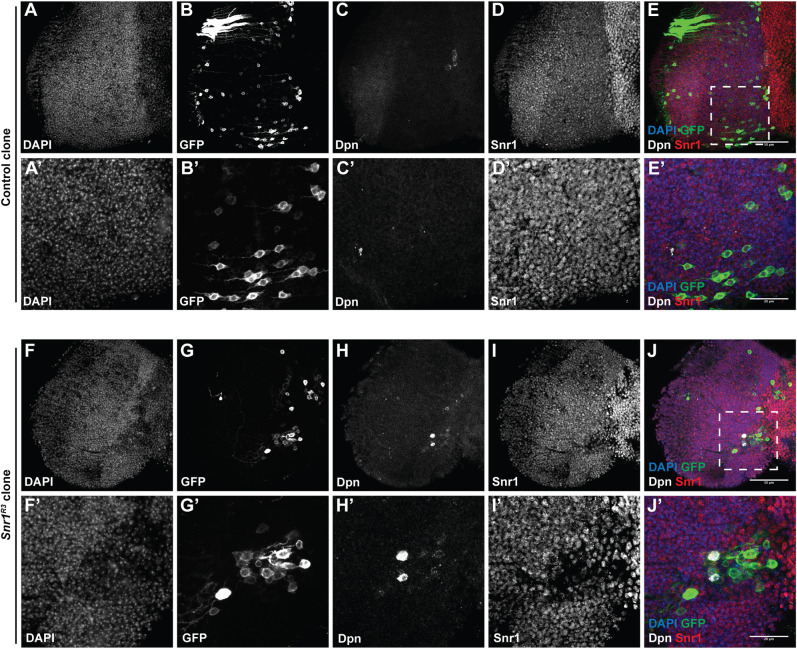
**Cells expressing the neuroblast marker Deadpan are present in *Snr1*^R3^ clones in the adult optic lobe.** Clones generated at 31 h after larval hatching (ALH). (A-E) Control MARCM clones marked with GFP. (A′-E′) Higher magnification images of region outlined in E. (F-J) *Snr1*^R3^ MARCM clone marked with GFP. (F′-J′) Higher magnification images of region outlined in J. In the merge, DAPI is in blue, GFP is in green, Dpn is in white and Snr1 is in red. Scale bars: 50 µm in E,J; 20 µm in E′,J′.

Given that *Snr1^R3^* mutant clones exhibit disrupted differentiation of neuroblasts with perdurance of neuroblast markers without triggering cell death, we investigated the fate of these *Snr1^R3^* mutant cells after larval development. Neuroblasts are normally eliminated by terminal differentiation or apoptosis before adulthood (Bello et al., 2003; Homem et al., 2014; Li et al., 2013; Maurange et al., 2008; Yang et al., 2017). We generated MARCM clones in 2nd instar larval brains and dissected adult brains 1 day after eclosion. Strikingly, we not only found that *Snr1^R3^* mutant clones persisted into the adult brain, but that they contained a small subset of Dpn^+^ cells (Fig. 4H,H′), whereas when wild-type clones were induced in 2nd instar larvae, no Dpn^+^ cells were detected in adult brains (Fig. 4C,C′). This was confirmed with *Snr1*^RNAi^ FLP-out clones, which similarly maintained Dpn^+^ cells in the adult brain (Fig. S3). The presence of Dpn^+^ cells at this stage indicated that cells from *Snr1*-deficient lineage clones generated during early larval development persisted throughout larval, pupal and early adult remodelling as undifferentiated neuroblasts.

### Single cell RNA sequencing of the brain identified differentially expressed Snr1 targets

We found Snr1 is needed for the transition from neuroepithelial cells into neuroblasts, as well as for neuroblast differentiation. These transitions are regulated by multiple pathways (Apitz and Salecker, 2014), and Snr1 has been shown to regulate the expression of a large number of genes (Tegeder et al., 2019). Comparative single cell RNA-sequencing (scRNA-seq) of the entire *Drosophila* central brain and optic lobes was used to identify the likely targets of Snr1 that are important for guiding these transitions (Fig. S4A). High levels of mRNA expression of known neuroepithelial or neuroblast identity genes were used to identify cell-type-specific clusters (Ashraf et al., 1999; Egger et al., 2010) (Fig. S4B,C). Optic lobe-specific clusters were re-clustered (Fig. S4D) and expression of neuroepithelial and neuroblast markers was used to confirm the cell types in the two clusters (Brunet Avalos et al., 2019; Dillon et al., 2022; Egger et al., 2010; Konstantinides et al., 2022) (Fig. S4E). To differentiate clusters containing optic lobe as opposed to central brain cells, we examined clusters for high levels of mRNAs encoding transcription factors that specifically pattern optic lobe medulla neuroblasts (Li et al., 2013). The transcription factors *homothorax* (*hth*), *eyeless* (*ey*), *sloppy paired 1* (*slp1*), *diachete* (*D*) and *tailless* (*tll*) were appropriately expressed in the neuroepithelial cell cluster and the neuroblast cluster in agreement with previous results (Konstantinides et al., 2022; Li et al., 2013), indicating that these clusters do represent cells from the optic lobe (Fig. S5).

**Fig. 5.**
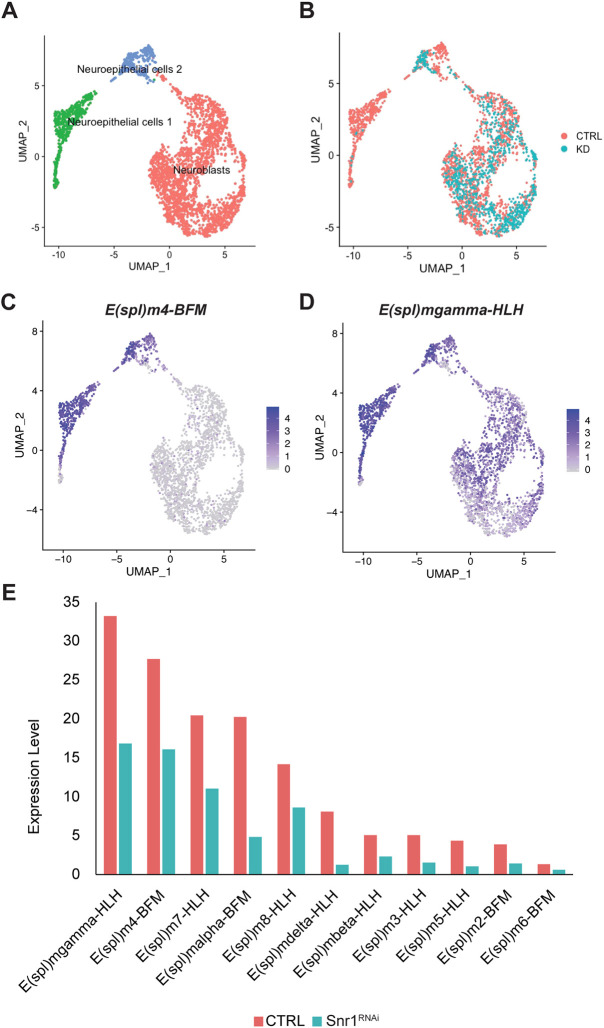
**Notch signalling is reduced in *Snr1* knockdown cells.** (A) Combined UMAP of optic lobe neuroepithelial cells and neuroblasts from control brains and brains with knockdown of *Snr1* in neuroepithelial cells. (B) UMAP showing distribution of optic lobe cells from the control brains (CTRL, red) and the *Snr1* knockdown brains (KD, blue). (C,D) UMAPs showing expression of Notch targets *E(spl)m 4-BFM* and *E(spl)mγ-HLH* in neuroepithelial cells and neuroblasts represented in A and B. (E) Average expression of *E(spl)* genes in control and the *Snr1* knockdown in neuroepithelial cells.

**Fig. 6.**
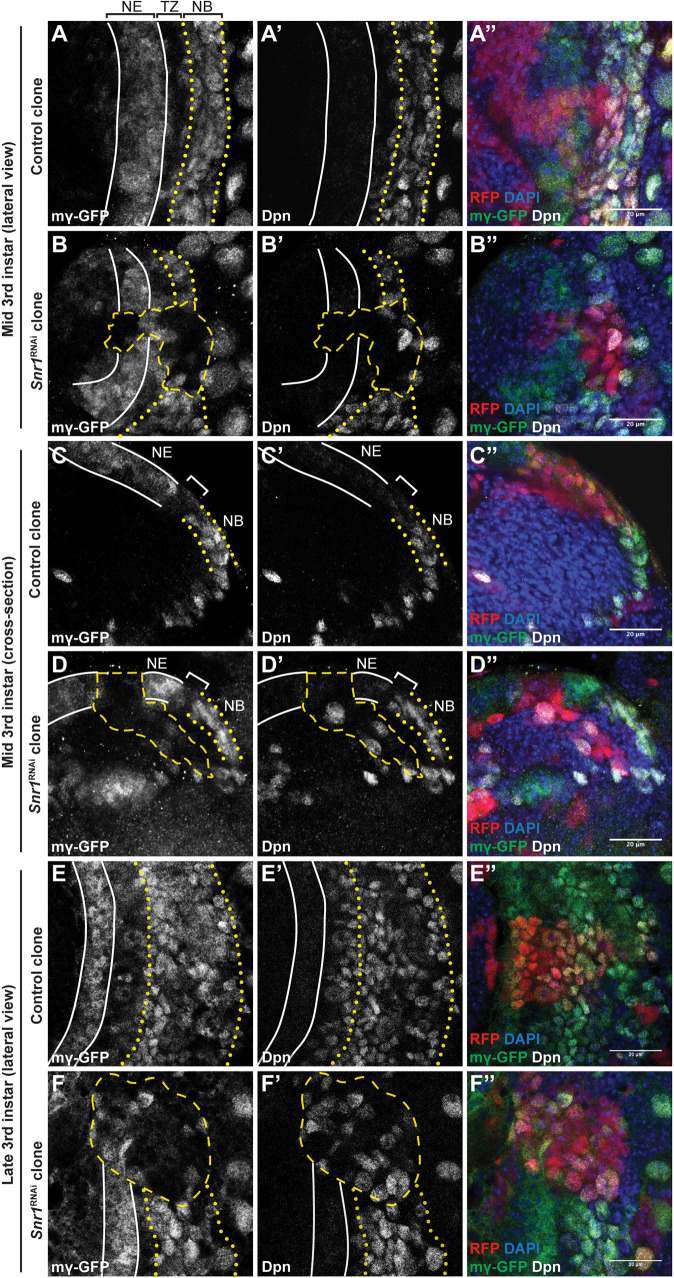
**E(spl)mγ expression is reduced in neuroepithelial and neuroblasts in *Snr1* knockdown cells.** (A-A″) *E(spl)mγ*-GFP expression in the optic lobe medulla at the mid 3rd instar stage. *E(spl)mγ*-GFP is expressed in neuroepithelial cells (NE, outlined with solid white lines). Expression is reduced in the transition zone (TZ) and re-expressed in neuroblasts (NB, outlined with yellow dotted lines). (B-B″) *Snr1*^RNAi^ clone (outlined with a yellow dashed line) has reduced *E(spl)mγ*-GFP and Dpn expression. (C-C″) *E(spl)mγ*-GFP expression at the mid 3rd instar stage in cross-section. (D-D″) The *Snr1*^RNAi^ clone prematurely extruded from neuroepithelium. (C,C′,D,D′) The transition zone is marked by the bracket. (E-E″) *E(spl)mγ*-GFP expression in late 3rd instar brain. (F-F″) *E(spl)mγ*-GFP expression in *Snr1* knockdown clone (outlined with a yellow dashed line). Merged images show GFP in green, Dpn in white, DAPI in blue and the clone in red. Scale bars: 20 µm.

To identify lineage-specific transcription regulated by Snr1, we performed differential scRNA-seq on larval brains expressing *Snr1*^RNAi^ in either neuroepithelial cells or in neuroblasts, which were compared with the control brains with a focus on optic lobe cells. Expression of *Snr1*^RNAi^ in neuroepithelial cells resulted in a uniform manifold approximation and projection (UMAP) plot with three clusters: two representing neuroepithelial cells and one representing neuroblasts (Fig. 5A). *Snr1*^RNAi^ cells were greatly reduced in the ‘neuroepithelial cells 1’ cluster (1.6% of cells in cluster), but were present in other clusters (30% in ‘neuroepithelial cells 2’, 38% in ‘neuroblast’ cluster) (Fig. 5B). To determine which neuroepithelial cells were most affected by *Snr1*^RNAi^, we queried genes expressed in the ‘neuroepithelial cells 1’ cluster. Among the mRNAs with the highest relative expression in this cluster compared with other cell type clusters in the dataset were Notch target genes of the *Enhancer of split* Complex [*E*(*spl*)*-C*] [(*E(spl)m4-BFM*, log2FC=3.63; *E(spl)mα-BFM*, log2FC=3.33; *E(spl)mδ-HLH*, log2FC=3.09; *E(spl)m3-HLH*, log2FC=2.56; *E(spl)mγ-HLH*, log2FC=2.44, *P*<1e-100] (representative plots are shown in Fig. 5C,D) (Bailey and Posakony, 1995; Lecourtois and Schweisguth, 1995). The two neuroepithelial cell clusters were then combined to measure differential gene expression between control cells and cells expressing *Snr1^RNAi^* (Table S1). The average expression of each *E(spl)* gene was reduced in neuroepithelial cells with reduced *Snr1* expression (Fig. 5E; Fig. S6A,E,J). Notch signalling in the optic lobe is required to maintain the neuroepithelium and to coordinate the transition into neuroblasts (Egger et al., 2010; Ngo et al., 2010; Wang et al., 2011). Thus, to further investigate the effect of Snr1 knockdown on Notch signalling, *Snr1*^RNAi^ clones were generated in flies that expressed an E(spl)mγ-GFP reporter (Almeida and Bray, 2005; Contreras et al., 2018). In the control brains, E(spl)mγ-GFP was expressed in neuroepithelial cells, suppressed in the transition zone and then expressed again in neuroblasts (Fig. 6A,C,E). The brains were dissected at either mid (Fig. 6A,C) or late (Fig. 6E) third instar stages, when the optic lobe medulla contains a higher proportion of neuroepithelial cells or neuroblasts, respectively. In mid third instar *Snr1*^RNAi^ clones, E(spl)mγ-GFP expression was reduced in neuroepithelial cells (Fig. 6B,D). When imaged in cross-section, these clones could be seen as being extruded from the neuroepithelium and prematurely expressing Dpn (Fig. 6D-D″). In late third instar *Snr1*^RNAi^ clones, E(spl)mγ-GFP expression was reduced in Dpn^+^ neuroblasts (Fig. 6F-F″). Dpn^+^ cells were located throughout the clone, including where neuroepithelial cells are expected (Fig. 6F′). To confirm that *Notch* or *E(spl)* genes were Snr1 targets, we undertook a rescue experiment where *Notch*, *E(spl)m4-BFM* or *E(spl)m7-HLH* were overexpressed in *Snr1*^RNAi^ clones and found that overexpression of *Notch* and *E(spl) m4-BFM* partially rescued the phenotype of *Snr1*^RNAi^ clones (Fig. S7C,E). These rescue clones did express ectopic Dpn, but they remained at the brain surface, indicating they were not extruded prematurely like *Snr1*^RNAi^ clones (Fig. S7A). Expression of *E(spl)m7-HLH,* however, did not rescue the *Snr1*^RNAi^ phenotype (Fig. S7G). As clones with reduced Snr1 expression in the neuroepithelium exhibited altered morphology and organization (Figs 2F,H, 6D-D″), we also examined the scRNA-seq for changes in expression of adhesion molecules. Expression of Cadherin family genes [*Cad99C*, *dachsous* (*ds*) and *fat* (*ft*)] increased when Snr1 was knocked down in neuroepithelial cells (Fig. S6B).

**Fig. 7.**
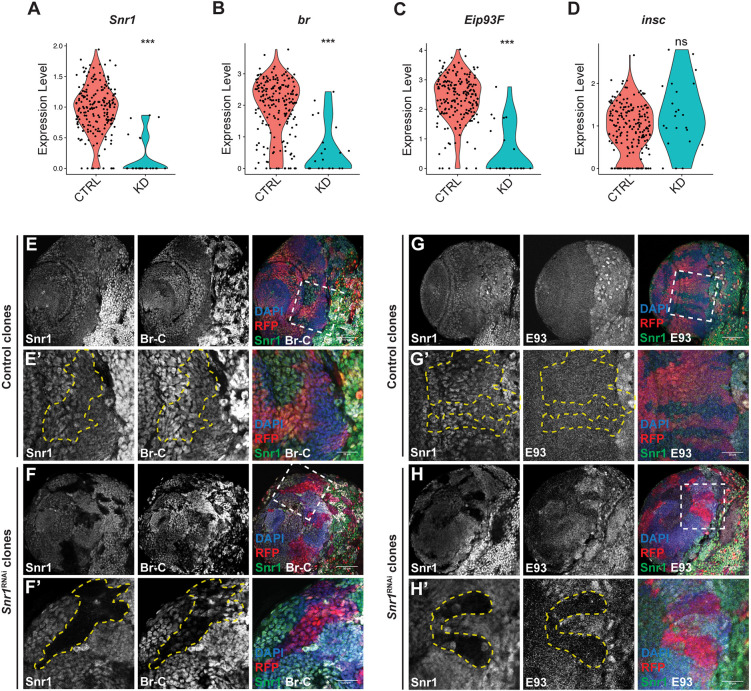
**Expression of *Broad* (*br*) and *Eip93F* is reduced in *Snr1* knockdown cells.** (A-D) Violin plots showing expression of *Snr1* (log2FC=-1.17, *P*=6e-11), *br* (log2FC=-1.94, *P*=1e-8), *Eip93F* (log2FC=-2.34, *P*=2e-11) and *inscuteable* (*insc*) (*P*=0.10) in neuroblasts from control brains and from brains expressing *Snr1*^RNAi^ in neuroblasts. ***P<0.001; ns, not significant. (E) FLP-out control clones. (E′) Higher magnification images of the region outlined in E. Clone is outlined by a yellow dashed line. (F) FLP-out clones expressing *Snr1*^RNAi^. (F′) Higher magnification image of the region outlined in F. Clone is outlined by a yellow dashed line. In merged images in E-F′, Snr1 is shown in green, Broad-Core (Br-C) is in white, RFP is in red and DAPI is in blue. (G) FLP-out control clones. (G′) Higher magnification image of region outlined in G. Clone is outlined by a yellow dashed line. (H) FLP-out clones expressing *Snr1*^RNAi^. (H′) Higher magnification image of region outlined in H. Clone is outlined by a yellow dashed line. In merged images in G-H′, Snr1 is shown in green, Eip93F (E93) is in white, RFP is in red and DAPI is in blue. Scale bars: 50 µm in E,F,G,H; 20 µm in E′,F′,G′,H′.

In contrast to *Snr1^RNAi^* in neuroepithelial cells, *Snr1*^RNAi^ in neuroblasts did not change the relative proportion of cells in each cluster. However, changes in mRNA levels were detected between wild-type and *Snr1*^RNAi^ neuroblasts (Fig. 7A). The strongest effects were on the mRNA of transcription factors: *broad* (*br*) (log2FC=-1.94, *P*=1e-8) and *Eip93F* (log2FC=-2.34, *P*=2e-11) (Fig. 7B,C; Table S2). Levels of the mRNA encoding neuroblast marker *insc*, however, were unchanged between the control and *Snr1*^RNAi^ cells (Fig. 7D). This change is not limited to the knockdown of Snr1 in neuroblast, as we found that knockdown of Snr1 in neuroepithelial cells also reduced the expression of *br* (log2FC=-0.57, *P*=1e-9) and *Eip93F* (log2FC=-0.38, *P*=9e-6) in these cells (Fig. S6K,L versus S6F,G). Additionally, the reduced expression levels of *Snr1*, *br* and *Eip93F* were maintained in neuroblasts that would be derived from these neuroepithelial cells (Fig. S6D,F,G,I,K,L). Thus, knockdown of Snr1 in either neuroepithelial cells or neuroblasts resulted in reduced expression of the Snr1 target genes *br* and *Eip93F.* Involvement of Eip93F in optic lobe neuroblast development has not previously been described; however, Br has been shown to be expressed in neuroepithelial cells and to promote their transition into neuroblasts (Zhou et al., 2019). Using *Snr1*^RNAi^ FLP-out clones, we analysed the effect on Br and Eip93F protein expression. Antibody detection of the Broad-core domain produced a robust signal in the optic lobe that was lost upon *Snr1*^RNAi^ (Fig. 7E,F). The Eip93F antibody signal was also reduced in *Snr1*^RNAi^ clones in the optic lobe (Fig. 7G,H). Given that *Snr1*^RNAi^ reduced levels of *br*, *Eip93F* and *Notch* pathway genes, we tested whether reducing expression of each gene would recapitulate the phenotype seen in *Snr1*^RNAi^ clones. Clones expressing *Notch*^RNAi^ or *br*^RNAi^ did resemble *Snr1*^RNAi^ clones with ectopic Dpn^+^ cells seen deep in the medulla (Fig. S8B-D); however, *Eip93F*^RNAi^ clones did not (Fig. S8E).

Analysis of the scRNA-seq of neuroblasts from brains with *Snr1*^RNAi^ in neuroepithelial cells also indicated a reduction in other transcription factors previously shown to pattern the brain, including several temporal transcription factors and one spatial transcription factor (*Visual system homeobox 1, Vsx1*) (Fig. S9A,C) (Erclik et al., 2017; Konstantinides et al., 2022; Li et al., 2013; Syed et al., 2017; Zhu et al., 2022). To determine whether differentiated neurons were also affected by *Snr1*^RNAi^ in neuroepithelial cells, our scRNA-seq was used to identify differentially expressed temporal markers of optic lobe neurons (Konstantinides et al., 2022; Li et al., 2013; Zhu et al., 2022). Of the series of known early, middle and late expressed neuronal markers, only a subset of middle and late markers had reduced expression in our dataset (Fig. S9B,D). Neuronal development was further assayed by generating *Snr1*^R3^ clones at the mid third instar stage to capture the transition from neuroblasts into neurons (Morante et al., 2011). Clones did not have cells with abnormal perdurance of Dpn expression (Fig. S9E,F), but did have altered morphology, appearing more clustered (Fig. S9F′) compared with the columnar organization of the control (Fig. S9E′), as previously described (Morante et al., 2011).

### Snr1 specifically targets brain development genes

**Fig. 8.**
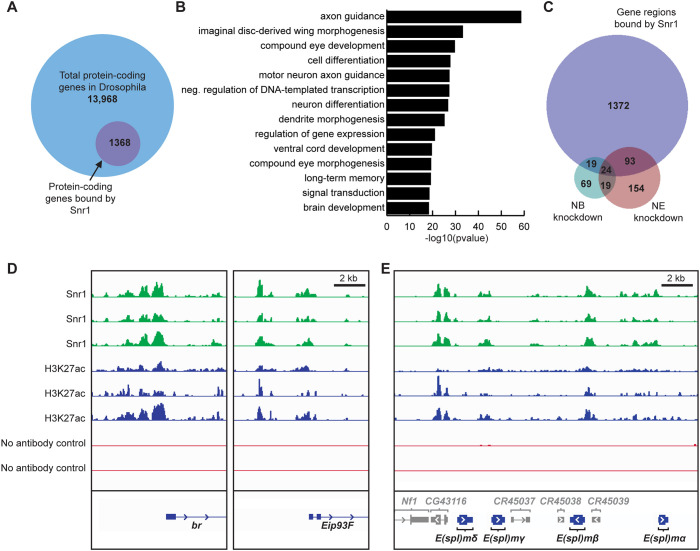
**Chromatin profiling reveals Snr1 occupancy at genes involved in brain development.** (A) Proportion of total protein-coding genes bound by Snr1. (B) Over-represented gene ontology terms associated with genes bound by Snr1. (C) Venn diagram comparing genes significantly downregulated in neuroblasts identified by scRNA-Seq with genes found in regions of chromatin bound by Snr1. Differentially expressed genes were identified from brains with *Snr1* knockdown in neuroepithelial cells (NE knockdown) or *Snr1* knockdown in neuroblasts (NB knockdown). (D) Chromatin profiling plots showing replicate experiments. Representative isoforms of *broad* (*br*) and *Eip93F* are shown. (E) Chromatin profiles of Enhancer of split complex genes.

As Snr1 is a subunit of the SWI/SNF chromatin remodelling complex (Dingwall et al., 1995), we next assayed whether changes in gene expression in the brain with *Snr1*^RNAi^ were a result of Snr1 targeting to these gene loci by Cleavage Under Targets and Tagmentation (CUT&Tag) (Kaya-Okur et al., 2019). This method inserts transposable elements in regions of chromatin associated with proteins of interest that are identified by sequencing. 1508 gene regions were strongly associated with Snr1 (top 10% of CUT&Tag targets; Table S3). These regions covered 1368 protein-coding genes, which represent 9.8% of protein-coding genes (Adams et al., 2000) (Fig. 8A). Over-representation analysis (ORA) of Snr1 targeted loci showed enrichment of genes involved in brain development, including axon guidance and neuron differentiation (Fig. 8B, Table S3). SWI/SNF complexes facilitate gene expression by promoting open chromatin (Schuettengruber et al., 2017), so loci strongly associated with Snr1 were compared with differentially expressed genes identified by scRNA-seq. 136 gene regions containing genes that had reduced mRNA levels showed Snr1 occupancy (Fig. 8C). Of genes with relatively lower mRNA levels, 33% (43 out of 131) from the neuroblast *Snr1*^RNAi^ and 40% (117 out of 290) from the neuroepithelial *Snr1*^RNAi^ overlapped with elevated Snr1 chromatin occupancy (Fig. 8C). Finally, we looked at chromatin occupancy at the loci of differentially expressed genes of interest from our scRNA-seq experiments. In agreement with our scRNA-seq results, which found that expression of *br* and *Eip93F* were reduced upon *Snr1*^RNAi^ (Fig. 7B,C), Snr1 associated with the likely transcriptional start sites of both *br* and *Eip93F* (Fig. 8D). High levels of Snr1 occupancy were likewise detected near the 5′ regions of *slp1*, *slp2*, *Vsx1* and *D* (Fig. S10A), which had reduced expression after Snr1 knockdown (Fig. S9A), whereas Snr1 was not enriched near *Cad99C*, *ds* and *ft* (Fig. S10B), which had higher mRNA levels due to *Snr1*^RNAi^ (Fig. S6B). The region surrounding the *E(spl)* complex was enriched for Snr1 (Fig. 8E). Generally, genes encoding mRNAs that had reduced levels in *Snr1*^RNAi^ brains had Snr1 associated with their transcriptional start sites, which coincided with active histone H3K27ac at the same region (Fig. 8D,E; Fig. S10A). Genes that had a strong H3K27ac signal but low or no Snr1 signal were also identified. These represented more general cellular processes (Fig. S11C,D). Thus, Snr1 is associated with multiple genes required for the proper transition of neuroepithelial cells into neuroblasts and subsequent neuroblast differentiation.

## DISCUSSION

Transcription factors that govern neuronal specification have been identified for many cell types in the *Drosophila* brain (Doe, 2017), but how these are collectively regulated is not clear. Generation of a particular type of neuron cannot be fully explained by the expression of temporal and spatial transcription factors in the progenitor cells (Sen et al., 2019). Some patterning genes are expressed at distinct time points from the generation of neurons while still influencing the type of daughter cell generated, indicating transcriptional memory (Erclik et al., 2017; Konstantinides et al., 2022). It has been proposed that unique chromatin states exist in neuroblasts expressing the same transcription factors (Chen and Konstantinides, 2022; El-Danaf et al., 2023; Janssens et al., 2022; Rossi et al., 2021; Sen, 2023). Although there is support for a model of chromatin state heterogeneity, how these are generated and maintained remains an open question.

We propose that Snr1 contributes to establishing a chromatin state defining neuroepithelial cells and maintaining this state in neuroblasts. Snr1 is present throughout neuroepithelial and neuroblast cell development (Fig. 1E), where loss of Snr1 affected two processes: the transition from neuroepithelial cells to neuroblasts (Fig. 2), and subsequent neuroblast differentiation (Figs 3 and 4). *Snr1*^RNAi^ in neuroepithelial cells caused premature differentiation into neuroblasts, demonstrating a role for Snr1 in maintaining neuroepithelial identity (Fig. 2D-H‴). Notch signalling likely contributes to this phenotype as Notch mutant clones are prematurely extruded from the neuroepithelium and resemble *Snr1*^RNAi^ clones (Fig. S8B,C) (Egger et al., 2010). Supporting this model, expression of a Notch target gene, *E(spl)mγ-HLH* was disrupted by *Snr1*^RNAi^ both in mid or late third instar development (Fig. 6B,D,F), leading to precocious expression of neuroblast markers (Fig. 6D′,F′). Snr1 was also present at this *E(spl)* locus, among several others (Fig. 8E). In further support of Snr1 acting as a regulator of Notch signalling, we found that overexpression of *Notch* or *E(spl)m4-BFM* partially rescued the phenotype of *Snr1*^RNAi^ clones (Fig. S7C,E). This is consistent with reports of the SWI/SNF complex contributing to the transcriptional response of Notch signalling (Pillidge and Bray, 2019). Based on our findings, Snr1 is likely needed to maintain open chromatin at Notch target genes. As Notch signalling maintains the neuroepithelium (Egger et al., 2010), repression of Notch targets due to loss of Snr1 would trigger the premature transition to neuroblasts we observed.

Neuroblasts divide asymmetrically to maintain themselves and produce GMCs that differentiate. When Snr1 expression was lost in clones in neuroepithelial or neuroblast cells, inappropriate expression of cell type markers (PatJ, Dpn and Elav) occurred and clone morphology was altered (Figs 2C,E,H and 3C). *Snr1*^RNAi^ clones also had disrupted expression of Br and Eip93F in neuroblasts, which have been identified as temporal transcription factors in late stage type I and type II neuroblasts (Liu et al., 2015; Syed et al., 2017) (Fig. 7B,C). Br is also expressed temporally in the optic lobe neuroepithelium and activated during later stages of larval development (Zhou et al., 2019). Eip93F contributes to cell-cycle exit of central brain neuroblasts during pupal stage (Maurange et al., 2008; Pahl et al., 2019). Whether Br and Eip93F act as neuroblast temporal transcription factors remains unclear, as they have a uniform pattern in the optic lobe (Fig. 7E,G) compared with the concentric ring pattern of other optic lobe temporal transcription factors (Li et al., 2013). We did find that knockdown of *Br* recapitulated the Snr1 clone phenotype (Fig. S8D). Thus, Snr1 contributes to the regulation of expression of genes required for appropriate neuroblasts activity.

In the absence of Snr1, neuroblasts were retained inappropriately, did not differentiate properly and persisted in the adult brain (Fig. 4; Fig. S3). A number of optic lobe temporal and spatial transcription factors, highlighted in recently published single cell sequencing data sets, had relatively lower mRNA levels in *Snr1*^RNAi^ brains (Fig. S9) (Konstantinides et al., 2022; Zhu et al., 2022). Neurons in our scRNA-seq had reduced expression of middle to late neuronal markers, suggesting that neurogenesis was stalled at the middle temporal window, leading to a decrease in later expressed neuronal and neuroblast transcription factors. The stalling of the temporal series indicates that neuroblasts fail to reach maturity and may explain why Snr1-deficient neuroblasts fail to differentiate. *Snr1*^R3^ clones generated in the mid third instar stage after neuroblast identity has been established, however, did not lead to ectopic Dpn^+^ cells (Fig. S9F). This suggests that the altered neuroblast differentiation is a result of defective chromatin state establishment in neuroblasts, rather than the ongoing activity of Snr1 in neuroblasts as they differentiate. This may indicate a crucial window for Snr1 activity during the neuroepithelial to neuroblast transition that has implications for neuroblasts throughout their maturation and differentiation. Our chromatin profiling showed that approximately one third of genes with changes in mRNA levels in *Snr1^RNAi^* brains also had relatively higher levels of Snr1 associated with the gene loci (Fig. 8C). Thus, Snr1 appears to regulate genes required for the developing brain, stabilizing neuroblast fate by maintaining chromatin accessibility at temporal and spatial transcription factors, as well as their target genes (Fig. S10A). The mechanisms underlying how Snr1 is targeted to these loci is a compelling area for further study.

Changes to optic lobe development when Snr1 is knocked down were also the result of altered differentiation and slower entry into the cell cycle (Fig. 3H). This effect of Snr1 knockdown in the brain is different from *Drosophila* wing discs where overgrowth was observed (Xie et al., 2017). *Snr1*^RNAi^ in wing discs triggered apoptosis, and co-expression of anti-apoptotic P35 exacerbated overgrowth (Xie et al., 2017). The role for Snr1 is clearly different in neuroblasts, as co-expression of P35 and *Snr1*^RNAi^ in the optic lobe instead did not alter cell number (Fig. S2E). However, it is possible that loss of Snr1 is triggering cell death in a caspase-independent manner (Hay et al., 1994; Xu et al., 2001). This role of Snr1 is clearly tissue specific, given the different effects in brain and wing discs, and will be an important consideration in future studies of Snr1 function.

Loss of SMARCB1 (the human Snr1 homolog) is the hallmark genetic alteration leading to atypical teratoid rhabdoid tumours (AT/RT), which are highly aggressive central nervous system tumours affecting infants and young children (Frühwald et al., 2016). Expression profiling of SMARCB1-deficient AT/RT tumours suggests these arise from stem cells; however, there is limited understanding of tumour progression (Deisch et al., 2011; Venneti et al., 2011). In *Drosophila*, we found neuroblasts persist inappropriately when Snr1 is reduced early during brain development (Fig. 4; Fig. S3), supporting a model whereby AT/RT tumours are derived from stem cells. A diagnostic feature of AT/RT tumours is the presence of poorly differentiated, multi-lineage cells expressing glial, mesenchymal and epithelial markers (Nesvick et al., 2020). Similarly, loss of Snr1 in the optic lobe leads to cells expressing an abnormal mixture of neuroepithelial, stem cell and neural markers (Fig. 2C,E; Fig. 3C), providing a powerful model for understanding the mechanisms underlying AT/RT.

## MATERIALS AND METHODS

### *Drosophila* stocks

UAS-*Snr1^RNAi^* (y^1^ sc* v^1^ sev^21^; P{TRiP.HMS00363}attP2, BL32372), UAS-*Notch^RNAi^* (*y^1^ v^1^*; P{TRiP.HMS00015}attP2, BL33621), UAS-*broad^RNAi^* (*y^1^ v^1^*; P{TRiP.HMS00042}attP2, BL33641), UAS-*Eip93F^RNAi^* (y^1^ sc* v^1^ sev^21^; P{TRiP.HMC04773}attP40, BL57868), *w^1118^*; P{UAS-N^full^}6 (BL52309), w*; P{UAS-E(spl)m4-BFM.A}15.5 (BL26679), y^1^ w*; P{UAS-E(spl)m7-HLH.C}2 (BL26681), *insc*-GAL4 (w*; P{GawB}*insc^Mz1407^*, BL8751), C855a-GAL4 (*w^1118^*; P{GawB}*C855a*, BL6990), *ogre*-GAL4 (*w^1118^*; P{GMR30D02-GAL4}attP2, BL 47530), P{hsFLP}12, P{UAS-GFP.U}1, y^1^ w*; P{*tubP*-GAL4}LL7 P{neoFRT}82B P{*tubP*-GAL80}LL3/*TM6C, Sb^1^ Tb^1^* (BL86311), w*; P{UAS-p35.H}BH1 (BL5072), P{hsFLP}1, *w^1118^*; *Adv^1^/CyO* (BL5072), *w^1118^*; P{neoFRT}82B P{Ubi-mRFP.nls}3R (BL30555), w*; P{neoFRT}82B/*TM6C, Sb^1^ Tb^+^* (BL86313) and *w^1118^*; P{UAS-Stinger}2 (BL84277) were obtained from the Bloomington *Drosophila* Stock Centre. The FRT82B*Snr1^R3^* line has been described previously (Dingwall et al., 1995). The hsFLP;; Act-FRT-Stop-FRT-GAL4, UAS-RFP has been described by Valentino and Erclik (2022). *w^1118^* flies were used as a control. Flies were maintained at 25°C (unless otherwise noted) on the following food: 5 g/l agar, 75 g/l cornmeal, 32 g/l yeast, 90 g/l sucrose and 2.5 g/l methyl 4-hydroxybenzoate (Schwarz et al., 2014).

### Immunofluorescence

Brains were dissected from wandering third instar larvae in phosphate-buffered saline (PBS, 130 mM NaCl, 7 mM Na_2_HPO_4_⋅2H_2_0, 3 mM NaH_2_PO_4_ 2H_2_O) and fixed in 4% paraformaldehyde in PBS for 20 min. Tissues were washed three times in PBS and blocked with 1% normal donkey serum (Sigma, D9663) and 0.1% Triton X-100 (Thermo Fisher, A16046.AE) in PBS (PTN) for 1 h at room temperature. Tissues were incubated with primary antibodies diluted in PTN overnight at 4°C. Tissues were washed for 1 h in PTN at room temperature, then incubated with secondary antibodies diluted in PTN for 2 h at room temperature, washed for 30 min in PTN and incubated with 4,6-diamidino-2-phenylindole (DAPI, Thermo Fisher D1306) in PTN for 10 min. Tissues were washed four times with PBS and mounted in ProLong Gold (Invitrogen, P36934). For labelling of dying cells, the In Situ Cell Death Detection kit, Fluorescein was used (Roche, 11684795910) as previously described (Abeysundara et al., 2018).

Primary antibodies used were: rat anti-DE-Cadherin (1:200, DSHB, DCAD2), mouse anti-Prospero (1:200, DSHB MR1A), mouse anti-Discs large (1:500, DSHB 4F3), rat anti-Elav (1:500, DSHB 7E8A10), mouse anti-Broad-core (1:500, DSHB 25E9.D7), rabbit anti-PatJ (1:1000) (Bhat et al., 1999), guinea-pig anti-Miranda (1:1000) (Kim et al., 2009), rabbit anti-Snr1 (1:500, this study; see Fig. S1A,B for validation data), rat anti-Deadpan (1:100, Abcam 195173), guinea-pig anti-Deadpan (1:1000) (Caygill and Brand, 2017), mouse anti-HA (1:500, Sigma 9658), mouse anti-phospho-histone H3 (1:2000, Abcam 14955), guinea-pig anti-Eip93F (1:250) (Syed et al., 2017). Secondary antibodies raised in donkey and used at 1:2000 were: anti-mouse AlexaFluor 555 (A31570) and AlexaFluor 647 (A31571), anti-rat AlexaFluor 488 (A21208) and AlexaFluor 647 (A78947), anti-rabbit Alexa Fluor 488 (A21206), AlexaFluor 555 (A31572) and AlexaFluor 647 (A31573) and anti-rat AlexaFluor 405 Plus (A48268) (all from Invitrogen), and anti-guinea-pig Alexa Fluor 488 (706-545-148) and Alexa Fluor 647 (706-605-148) and anti-rat Alexa Fluor 647 (712-606-153) (all from Jackson ImmunoResearch).

### 5-ethynyl-2′-deoxyuridine (EdU) labelling

EdU labelling was performed using Click-iT EdU Alexa Fluor 488 Imaging Kit (Invitrogen, C10337) as per the manufacturer's instructions. Brains from wandering third instar larvae were dissected in Schneider's media (Gibco 21720-024, serum-free) and incubated in 30 μM EdU in Schneider's media for 30 min at 25°C.

### Generation of Snr1 antibody

The *Snr1* open reading frame (cDNA clone GH08712, DGRC) was cloned into pENTR-D (Thermo Fisher) and transferred to pDEST-17 (Invitrogen). BL21-AI *Escherichia coli* (Thermo Fisher C6070-03) were transformed with plasmid DNA and grown to an OD_600_ of 0.4 at 37°C. Protein expression was induced with 0.2% L-arabinose (Sigma) at 25°C for 3 h. The cell pellet was lysed by incubation in 8 M urea (Sigma) in 1×PBS and a cleared lysate prepared and applied to a 1 ml HisTrap column (Cytavia 17524701), using a AKTA-Start Purification System. Purified protein was eluted with a stepwise imidazole gradient. Fractions containing purified Snr1 were pooled and desalted overnight by buffer exchange, concentrated to 1 μg/μl using Amicon Ultra 15 centrifugal filter unit (MilliporeSigma UFC901008 MWCO 10 Da) and injected into rabbits by Pocono Rabbit Farms and Laboratories (Canadensis, PA, USA). Affinity purification was performed by cloning Snr1 into pDEST-15 (Invitrogen) to express GST-tagged Snr1, which was cross linked to Glutathione Sepharose 4B beads (Cytavia 17075601) with 100 mM Dimethyl Pimelimidate (Sigma D8388). Antibody was validated by immunofluorescence and western blot on *Drosophila* larval brain (Fig. S1A,B).

### SDS-PAGE and western blotting

Ten third instar larval brains per well were run on 10% SDS-PAGE gel and transferred to nitrocellulose membrane. Membranes were imaged on the Odyssey Infrared Imaging System (LI-COR). Primary antibodies used were mouse anti-β-tubulin (1:3000, DSHB E7), rabbit anti-SNF5 (1:3000, Abcam ab126734) and rabbit anti-Snr1 (1:1000, this study). Secondary antibodies used were donkey anti-rabbit Alexa Fluor 680 (1:10,000, Jackson ImmunoResearch) and donkey anti-mouse Alexa Fluor 790 (1:25,000, Abcam).

### Lineage tracing

Somatic clones were generated using FLP/FRT (Golic and Lindquist, 1989; Xu and Rubin, 1993). Larvae were heat shocked at 37°C for 30 min at the early 2nd instar stage [31 h after larval hatching (ALH)] when the optic lobe is largely composed of neuroepithelial cells (Egger et al., 2007), unless otherwise noted (Fig. 2A). Clones were identified by fluorescent markers (GFP or RFP) in late third instar larval optic lobes (96 h ALH) after most neuroepithelial cells have transitioned into neuroblasts unless otherwise noted. Mid third instar clones were dissected at 72 h ALH when a larger proportion of neuroepithelial cells are present (Figs 2G-H‴ and 6A-D″). Neuroblast clones were heat shocked at 72 h ALH and dissected at 96 h ALH (Fig. S9E-F″).

### Microscopy

Imaging was performed using a Zeiss LSM 700 confocal microscope using a 20× NA 0.8 objective (Plan-Apochromat) and a 40× NA 1.3 oil immersion objective (EC Plan-Neofluar). The acquisition software used was Zen 2009. ImageJ and Adobe Illustrator 27.0 were used to assemble the figures. Fig. 1A,B were created with BioRender.com. Quantification of cell number, FI^+^ cells, clone volume and pH3^+^ cells was carried out in Imaris 9.6 (Bitplane).

### HA-Snr1 expressing flies

An oligonucleotide encoding a triple HA-tag (TACCCATACGATGTTCCTGACTATGCGGGCTATCCCTATGACGTCCCGGACTATGCAGGATCCTATCCATATGACGTTCCAGATTACGCT) was cloned into the N-terminal end of the full-length *Snr1* open reading frame by restriction enzyme cloning. Snr1 was transferred into pUASg-attB (DGRC) and injected into embryos for PhiC31-mediated integration at the attP40 site by BestGene.

### qRT-PCR

Ten wandering third instar larval brains were collected in PBS for each genotype. Samples were flash frozen in liquid nitrogen and RNA extraction was carried out using Trizol (Life Technologies 15596026). cDNA synthesis was performed with the Maxima H minus cDNA synthesis Master Mix with dsDNase (Thermo Scientific M1681). Quantitative PCR was performed in triplicate using an Eppendorf MasterCycler RealPlex2 with Perfecta SYBR Green FastMix (QuantaBio 95118). Expression was normalized to *Ribosomal protein L30* (*RpL30*) mRNA using the ΔΔCt method (Schmittgen and Livak, 2008). *Snr1* primers were: forward, AGCTATCGTGGCACTGTCGAAC; reverse, TTCCGGAAGGGCACATCAATCG. *Rpl30* primers were: forward, GGTGCACACGCGGAAGTATT; reverse, GCCCTGAGGAAGTCCGAGAT.

### Single cell sequencing sample preparation

Twenty wandering third instar larval brains per genotype were dissected in PBS. Attached tissues and ventral nerve cords (VNCs) were removed. Dissection time was less than 30 min and samples were on ice for under 30 min before dissociation. All steps were performed using siliconized microfuge tubes and pipette tips (Sigmacote, Sigma SL2). Dissociation solution was made by dissolving 1 mg/ml collagenase (Sigma C2674) in 1×PBS. Immediately before use, 5 mM CaCl_2_ (EMD) was added to collagenase solution (1:100 from 500 mM stock solution). Brains were incubated in 200 μl of the dissociation solution at 25°C in a shaking heat block (500 rpm) for 30 min pipetting the brains up and down every 10 min to increase dissociation. Another 200 μl of the dissociation solution was added and incubated again as above. Dissociation was stopped by adding 1 ml PBS with 0.04% bovine serum albumin (BSA) (Sigma A7906). Individual cells were isolated by passage through 40 μm sterile cell strainer (ThermoFisher 22363547) and pelleted by two centrifugations at 1000 ***g*** for 5 min at 4°C. Cell pellets were transferred to 50 μl PBS with 0.04% BSA and dispersed by pipetting up and down 15 times. Cell density of a 5 μl sample of cell solution was obtained by Trypan Blue staining and counting on a haemocytometer. Samples were diluted to 1000 cells/μl in PBS with 0.04% BSA. For the neuroblast *Snr1^RNAi^*, the Dead Cell Removal Kit (Miltenyi Biotec, 130-090-101) was used.

### Single cell sequencing

Single cell RNA sequencing libraries were prepared as per Chromium Next GEM single Cell 3′ Kit v3.1 (10X Genomics) with a target of 10,000 cells per sample. Eight libraries were sequenced by paired-end sequencing using the NovaSeq6000 system, PE 150 (Novogene) averaging 50,000 reads per cell.

### Single cell sequencing analysis

Cell Ranger (10X Genomics) was used to generate feature-count matrices aligned to the BDGP6.32 genome assembly. Analysis was performed in R (version 4.1.1) using the Seurat package (Seurat V4) according to Hao et al. (2021). Raw count data were combined for quality control processing. For neuroblast *Snr*1 knockdown, cells were filtered to include only cells with between 700 and 5000 features per cell and less than 6% mitochondrial reads. For neuroepithelial *Snr1* knockdown, cells were filtered to select cells with between 700 and 4500 features per cell and less than 5% mitochondrial reads. Control and knockdown datasets were normalized separately and the top 2000 variable features identified. These were used to merge the datasets using the IntegrateData function. The integrated data were scaled and principal component analysis was performed. Significant principal components (PCs) were determined (ElbowPlot). The top 20 PCs were used to cluster cells at a resolution of 0.55 and UMAP plotting (RunUMAP). The FindAllMarkers function was used to identify clusters containing neuroblasts and neuroepithelial cells. Plots showing expression levels of specific genes were generated with FeaturePlot. Genes differentially expressed between the control and the knockdown experiments in each cluster were identified using FindMarkers. Cells expressing *optic ganglion reduced* (*ogre*) were used to subset optic lobe cells, which were re-clustered (Fig. S11A). Ogre is expressed throughout the entire optic lobe, as shown by GFP expression (Fig. S11B).

### Statistical analysis

At least three biological replicates were performed for each experiment, except the scRNA-seq experiment, which was performed once. For scRNA-Seq data, the non-parametric Wilcoxon rank sum test was used for differential expression analysis. For comparison between groups in Fig. S2G-I, the Kruskal–Wallis test was used. All other *P*-values were calculated using the Wilcoxon rank sum test. Boxes in box plots represent the interquartile range (IQR). Whiskers extend to nearest data point within 1.5 IQR of box. Mean±s.d. is reported in the figure legends.

### CUT&Tag

Chromatin profiling was performed using CUT&Tag-IT (Active Motif, 53160). 25 *w^1118^* wandering third instar larval brains with VNC removed were dissected in PBS and collected in a silicone-coated tube on ice. Tissues were dissociated and nuclei extracted as per Janssens et al. (2022), resuspended in 500 μl nuclei lysis buffer [10 mM Tris-HCl (pH 7.4), 10 mM NaCl, 3 mM MgCl, 0.1% Tween-20, 0.1% Nonidet P40, 0.01% Digitonin, 1% BSA and 1× Roche Complete EDTA free protease inhibitor tablet], transferred to 1 ml glass dounce tissue grinder and incubated on ice for 5 min. The loose pestle was applied 25 times followed by incubation on ice for 10 min, and then the tight pestle was applied 25 times. Nuclei wash buffer [1 ml; 10 mM Tris-HCl (pH 7.4), 10 mM NaCl, 3 mM MgCl, 0.1% Tween-20, 1% BSA and 1× Roche Complete EDTA free protease inhibitor tablet) was added to the final lysate and transferred to a 1.5 ml microfuge tube. After centrifugation at 1500 ***g*** for 5 min at 4°C, the supernatant was removed and nuclei resuspended in 300 μl 1× Wash Buffer with Protease Inhibitor Cocktail (Active Motif), passed through 40 μm cell strainer (ThermoFisher 22363547) and spun down twice at 1500 ***g*** for 5 min at 4°C. The supernatant was removed and 1.5 ml wash buffer was added. The resulting nuclei suspension was divided between three tubes (500 μl each) and 1 ml wash buffer added to each. Concanavalin A bead slurry (20 μl) was added to each tube and CUT&Tag performed as per the Active Motif CUT&Tag-IT Assay Manual (Version A4). 1μg of rabbit anti-SNF5 (0.015 μg/μl, Abcam ab126734, ChIP-grade and cross-species validated, Fig. S1D) and 1μg rabbit anti-Histone H3K27ac (0.02 μg/μl, Active Motif 39034) were used. Negative controls omitted the primary antibody. The quality of the library was assessed for DNA concentration (Qubit) and for average fragment size by High Sensitivity DNA assay (Agilent 2100 bioanalyzer). Sequencing was performed on the Illumina NextSeq system (Molecular Biology Service Unit, University of Alberta).

Sequencing results were processed according to Zheng et al. (2020) and Henikoff et al. (2020). Scaling factors were normalized by the ChIPseqSpikeInFree method. Results were filtered to include the top 10% of signal identified by Sparse Enrichment Analysis (Meers et al., 2019) and visualized using the Integrated Genomics Viewer (Broad Institute) (Robinson et al., 2011). Genes within 1 kb of *Snr1* peaks were considered potential Snr1 targets. ORA was performed with easyGSEA (Cheng et al., 2021). Venn diagrams were produced using BioVenn (Hulsen et al., 2008).

## Supplementary Material

10.1242/develop.201484_sup1 Supplementary information
