# Supplementary material for: A chromatin remodelling SWI/SNF subunit, Snr1, regulates neural stem cell determination and differentiation
Source: Development. 2023 Jun 30;150(13):dev201484. doi: 10.1242/dev.201484 (PMC10323235; doi:10.1242/dev.201484)
Supplement: Supplementary information [file develop-150-201484-s1.pdf]

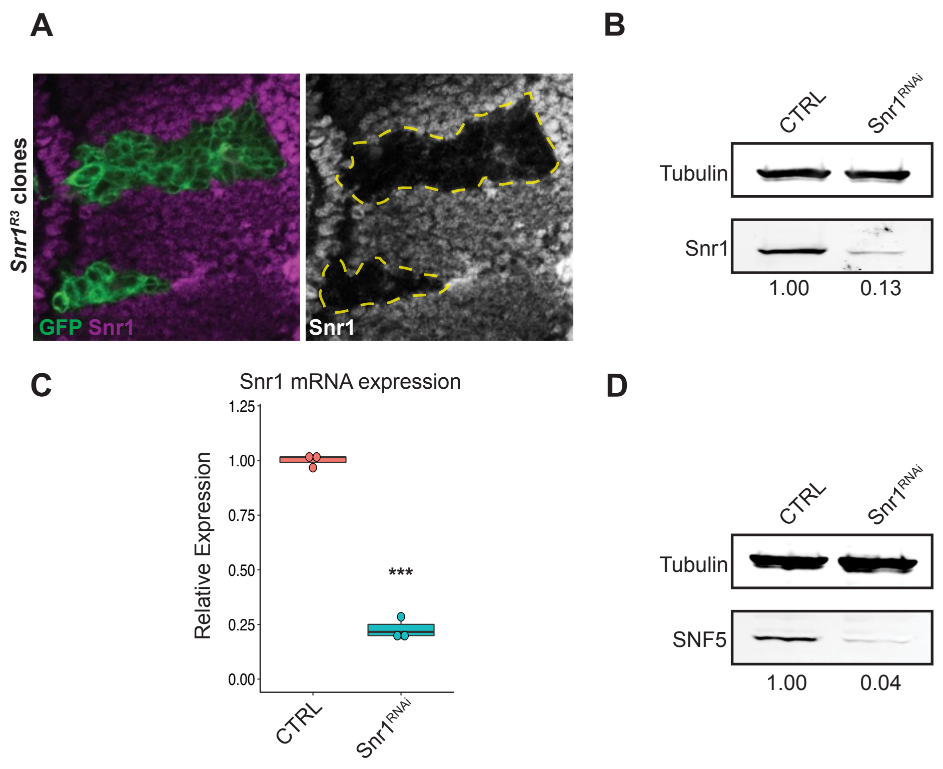

Fig. S1. Validation of Snr1 antibodies and RNAi knockdown efficiency. A) *Snr1*^{R3} MARCM clones (outlined with yellow dashed line) in the optic lobe marked by GFP (green). Snr1 marked by an antibody generated in this study (magenta). B) Snr1 protein expression in control brains (CTRL) and in brains expressing *Snr1*^{RNAi} in neuroblasts (KD) (*insc>Snr1*^{RNAi}). Snr1 levels (labelled with Snr1 antibody) normalized to Tubulin (n=3, 0.13±0.06, p=1e-5). C) Relative *Snr1* RNA expression in control brains (CTRL) (n=3, 1.00±0.03) and brains expressing *Snr1*^{RNAi} in neuroblasts (KD) (n=3, 0.23±0.05). P=2e-4. *** = p<0.001. D) Snr1 protein expression in control brains (CTRL) and brains expressing *Snr1*^{RNAi} in neuroblasts (KD). Snr1 levels (labelled with SNF5 antibody) normalized to Tubulin (n=3, 0.04±0.02, p=5e-8).

Development • Supplementary information

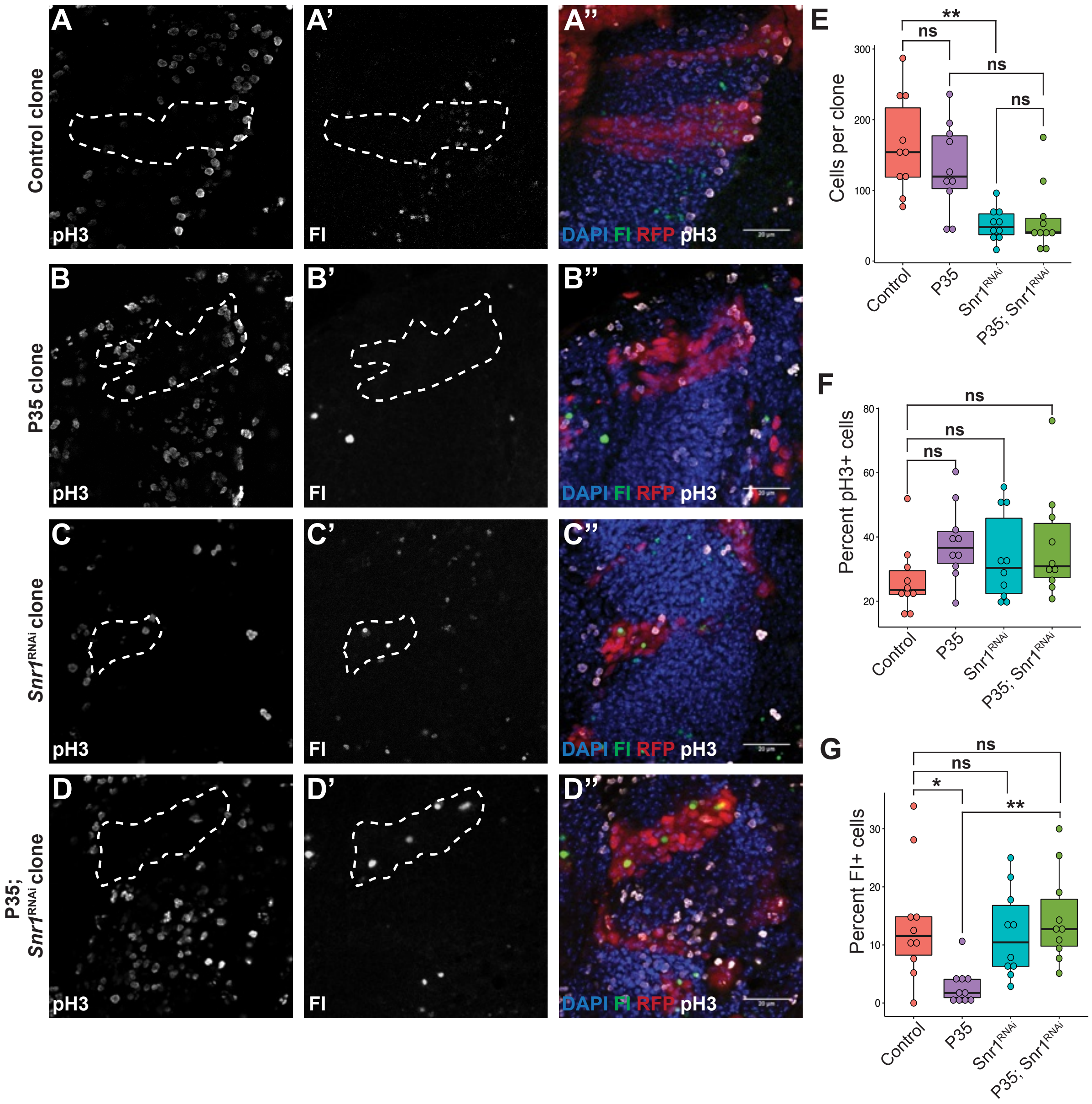

Fig. S2. Mitotic cell number and apoptosis are not affected by Snr1 knockdown. A-A’’) Control FLP-out clone. B-B’’) FLP-out clone expressing P35. C-C’’) FLP-out clone expressing *Snr1*^{RNAi}. D-D’’) FLP-out clone expressing P35 and *Snr1*^{RNAi}. A-D’’) Dying cells labelled with fluorescein (FI). Mitotic cells labelled with phospho-histone H3 (pH3). Clones outlined with white dashed lines. Scale bar = 20 µm. E) Cells per clone in control (n=10, 164± 69), *Snr1*^{RNAi} (n=10, 52± 23), P35 (n=10, 132± 63), and P35;*Snr1*^{RNAi} (n=10, 60± 49) clones. p=2e-4. F) Percent mitotic cells identified based on pH3 signal in control (n=10, 27± 11%), *Snr1*^{RNAi} (n=10, 34± 14%), P35 (n=10, 38± 12%), and P35;*Snr1*^{RNAi} (n=10, 37± 17%) clones. p=0.13. G) Percent dying cells based on FI signal in control (n=10, 14± 10%), *Snr1*^{RNAi} (n=10, 12± 8%), P35 (n=10, 3± 3%), and P35;*Snr1*^{RNAi} (n=10, 15± 8%) clones. n=10. p=1e-3.

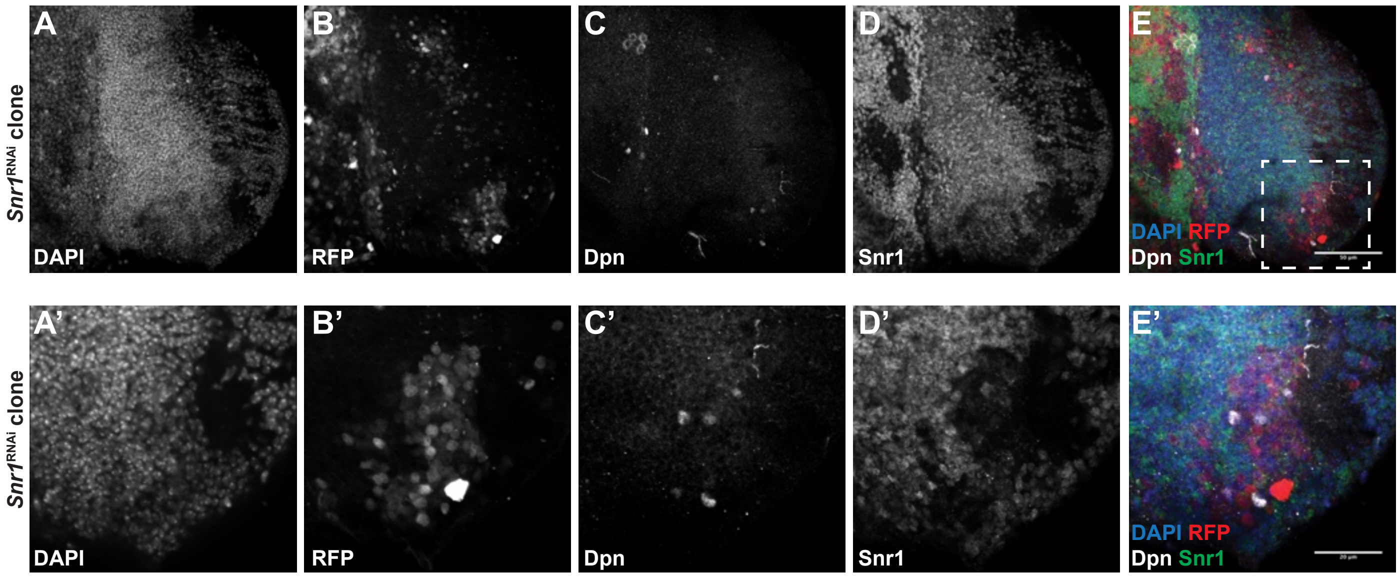

Fig. S3. *Snr1*^{RNAi} clones express neuroblast marker Deadpan in the adult optic lobe. A-E) FLP-out *Snr1*^{RNAi} clone in the adult brain. Clone labelled with RFP. Neuroblasts labelled with Dpn. Scale bar = 50 µm. A'-E') Higher magnification of region outlined with white dashed line in E. Scale bar = 20 µm.

Development • Supplementary information

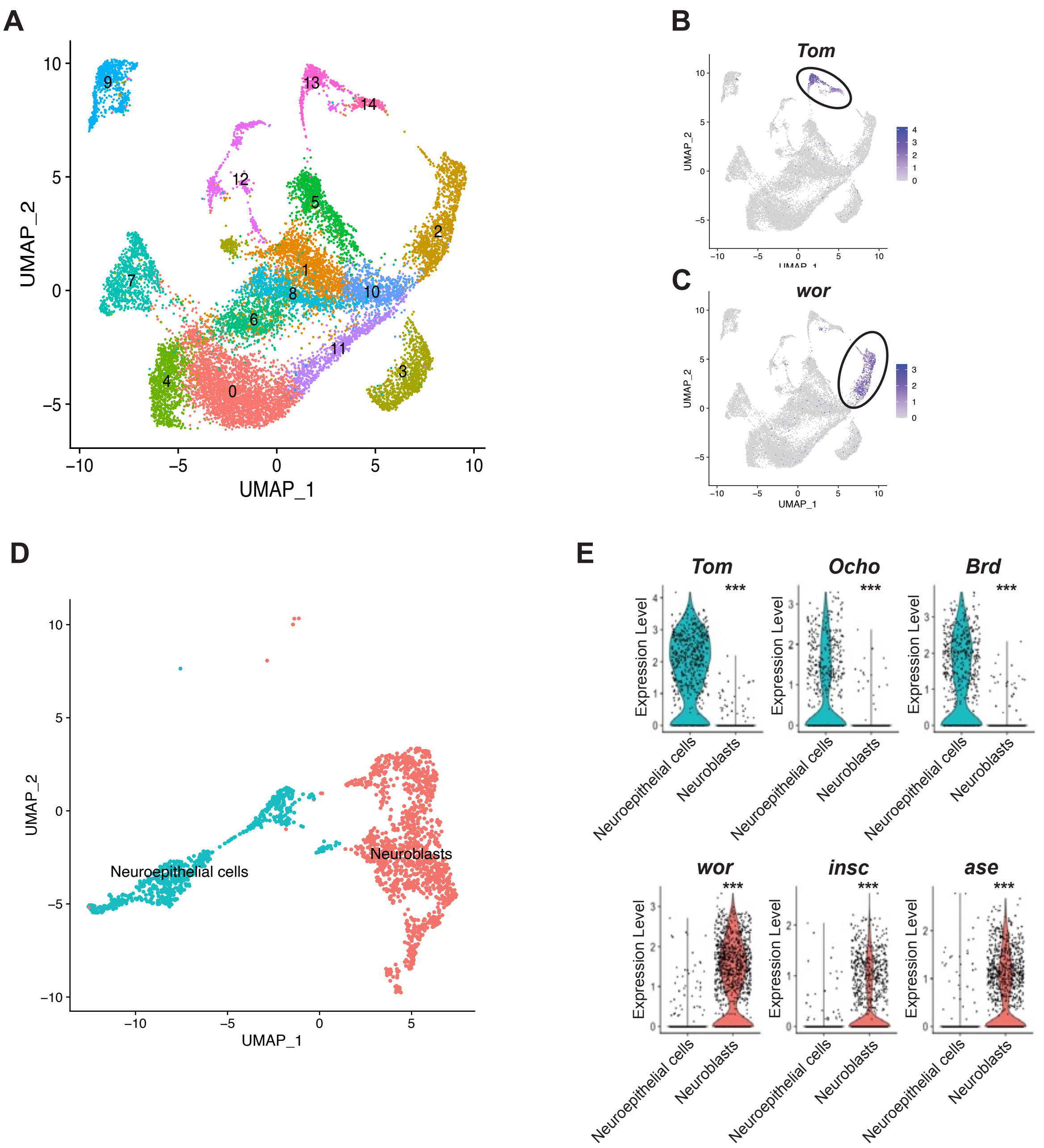

Fig. S4. Identification of optic lobe neuroepithelial cells and neuroblasts by single cell sequencing. A) UMAP of whole larval brain. B) Expression of neuroepithelial cell marker *Twin of m4 (Tom)*. C) Expression of neuroblast marker *worniu (wor)*. B-C) Encircled cells used for re-clustering in D. D) Re-clustering of neuroepithelial cells (blue) (n=648) and neuroblasts (red) (n=1267). E) Expression in clusters shown in D of neuroepithelial markers *Tom* (log2FC=-3.09, p=5e-291), *Ocho* (log2FC=-1.81, p=5e-137), *bearded* (*Brd*) (log2FC=-2.47, p=3e-189). Expression in clusters shown in D of neuroblast markers *worniu* (*wor*) (log2FC=1.92, p=2e-133), *insc* (log2FC=-0.82, p=4e-56), *asense (ase)* (log2FC=1.05, p=1e-84). ***=p<0.001.

Development • Supplementary information

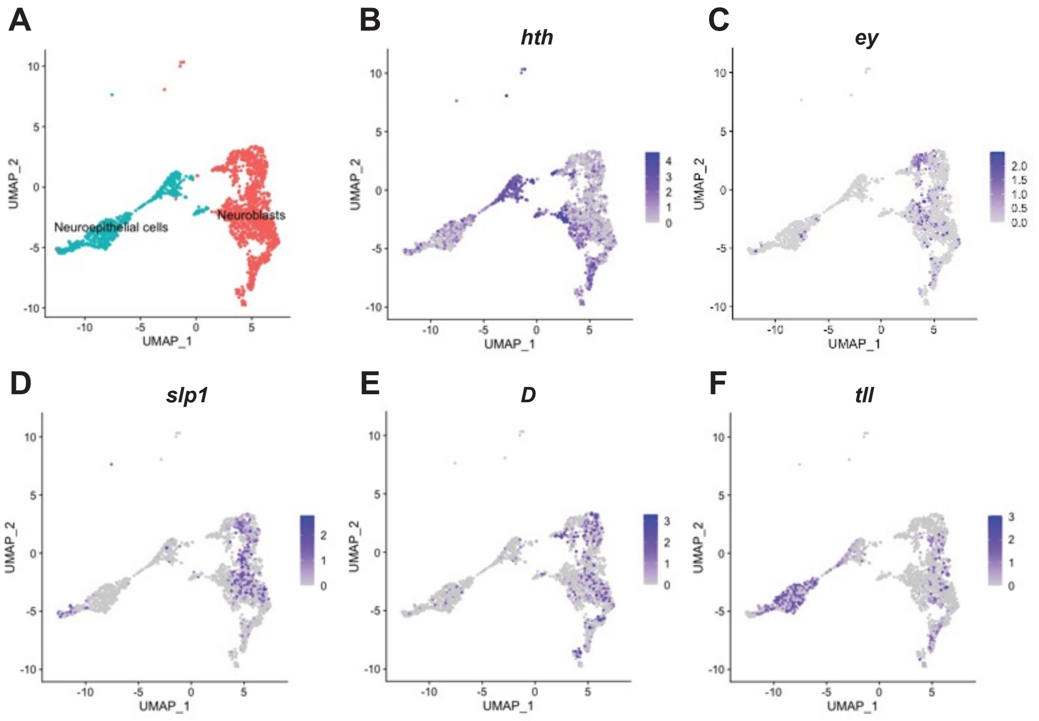

Fig. S5. Expression of optic lobe temporal transcription factors. UMAPs showing expression levels in neuroepithelial cells and neuroblasts. A) UMAPs show cells represented in Fig. S4D. B) Expression of *homothorax* (*hth*), C) *eyeless* (*ey*), D) *sloppy paired 1* (*slp1*), E) *Diachete* (*D*), and F) *tailless* (*tll*).

Development • Supplementary information

A

Gene	Average Log2 Fold Change	P-value
E(spl)malpha-BFM	-1.871	1.68e-25
E(spl)mdelta-HLH	-2.009	5.45e-21
E(spl)m3-HLH	-1.262	3.00e-15
E(spl)m5-HLH	-1.381	5.10e-13
E(spl)mgamma-HLH	-0.938	9.28e-12
E(spl)m7-HLH	-0.836	1.08e-09
E(spl)m8-HLH	-0.657	1.77e-08
E(spl)m2-BFM	-1.026	1.61e-07
E(spl)m4-BFM	-0.750	2.22e-07
E(spl)mbeta-HLH	-0.875	2.26e-07
E(spl)m6-BFM	-0.554	6.31e-04

B

Gene	Average Log2 Fold Change	P-value
Cad99C	0.458	1.82e-24
ds	0.738	2.75e-10
ft	0.770	1.91e-09

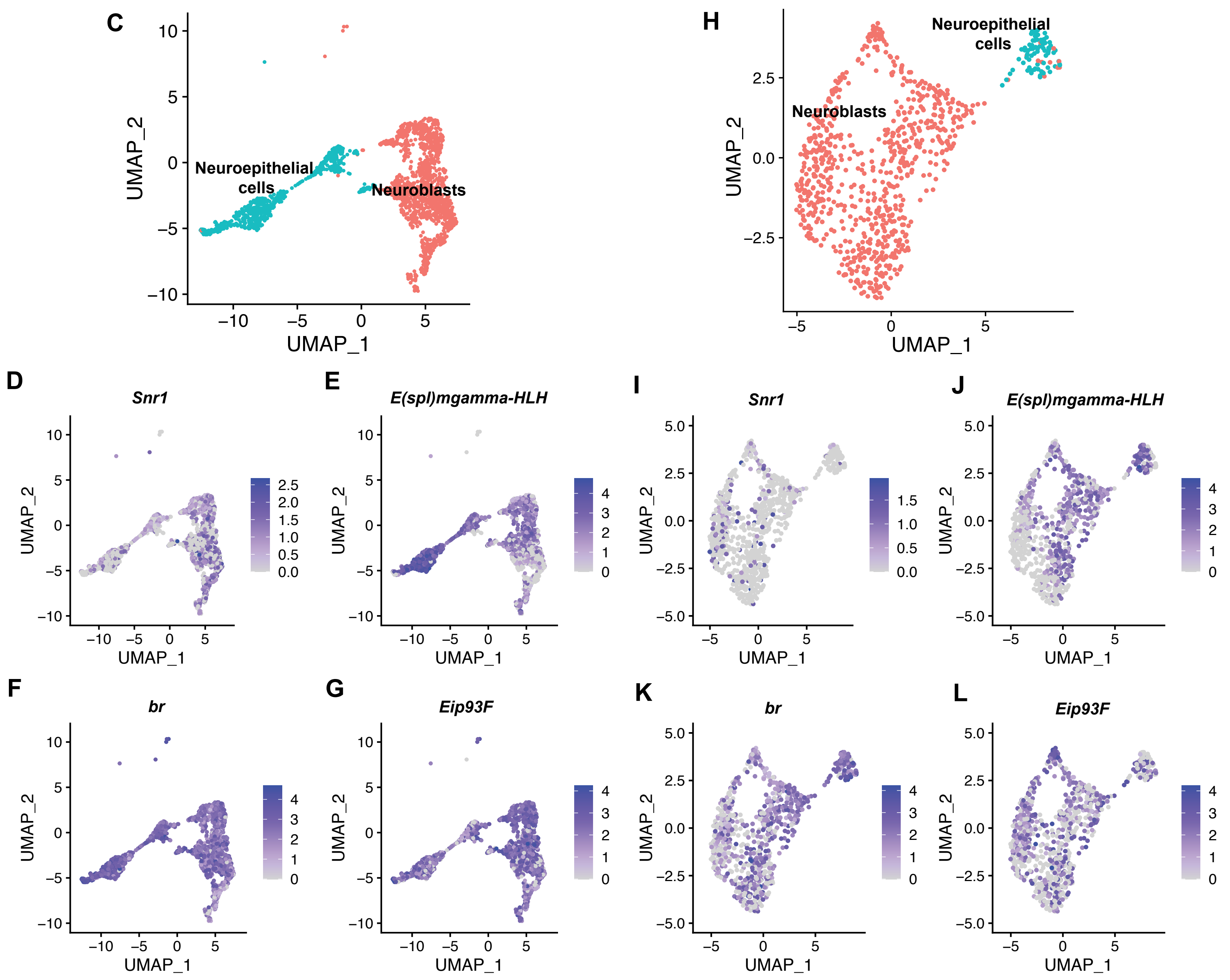

Development • Supplementary information

Fig. S6. Differential mRNA levels of genes in *Snr1* knockdown in neuroepithelial cells. A) Differential expression of *E(spl)* genes. B) Differential expression of adhesion genes. C) UMAP of control optic lobe cells. D-G) UMAPs showing gene expression in cells shown in C. H) UMAP of optic lobe cells from brains expressing *Snr1*^{RNAi} in neuroepithelial cells. I-L) UMAPs showing gene expression in cells shown in H.

Development • Supplementary information

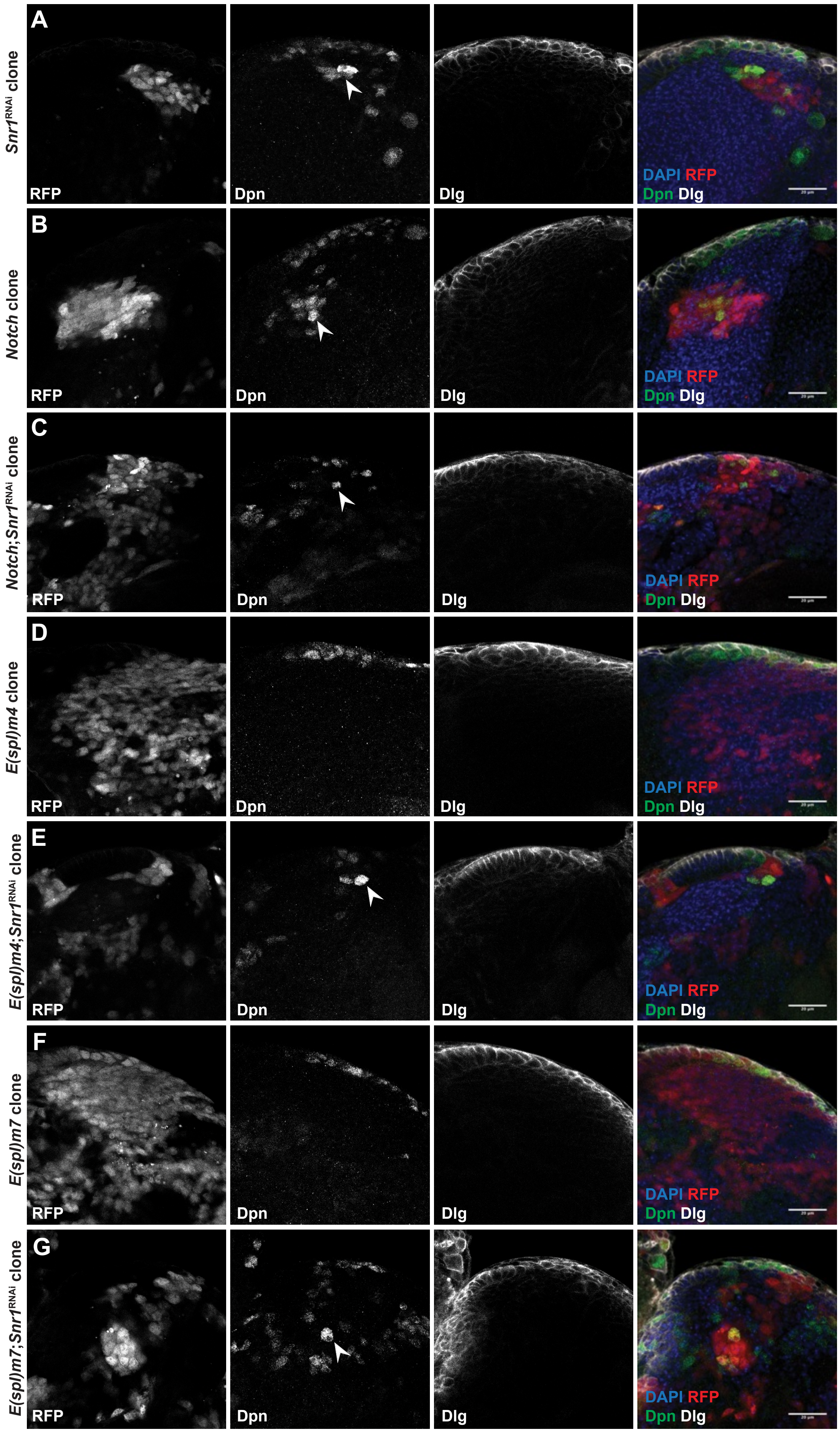

Fig. S7. Over-expression of Notch pathway genes partially rescues Snr1 knockdown phenotype. FLP-out clones expressing A) *Snr1*^{RNAi}, B) UAS-*Notch*, C) UAS-*Notch*; *Snr1*^{RNAi}, D) UAS-*E(spl)m4-BFM*, E) UAS-*E(spl)m4-BFM*; *Snr1*^{RNAi} , F) UAS-*E(spl)m7-HLH*, and G) UAS-*E(spl)m7-HLH*; *Snr1*^{RNAi}. A-G) Clones marked by RFP. Scale bars = 20 µm.

Development • Supplementary information

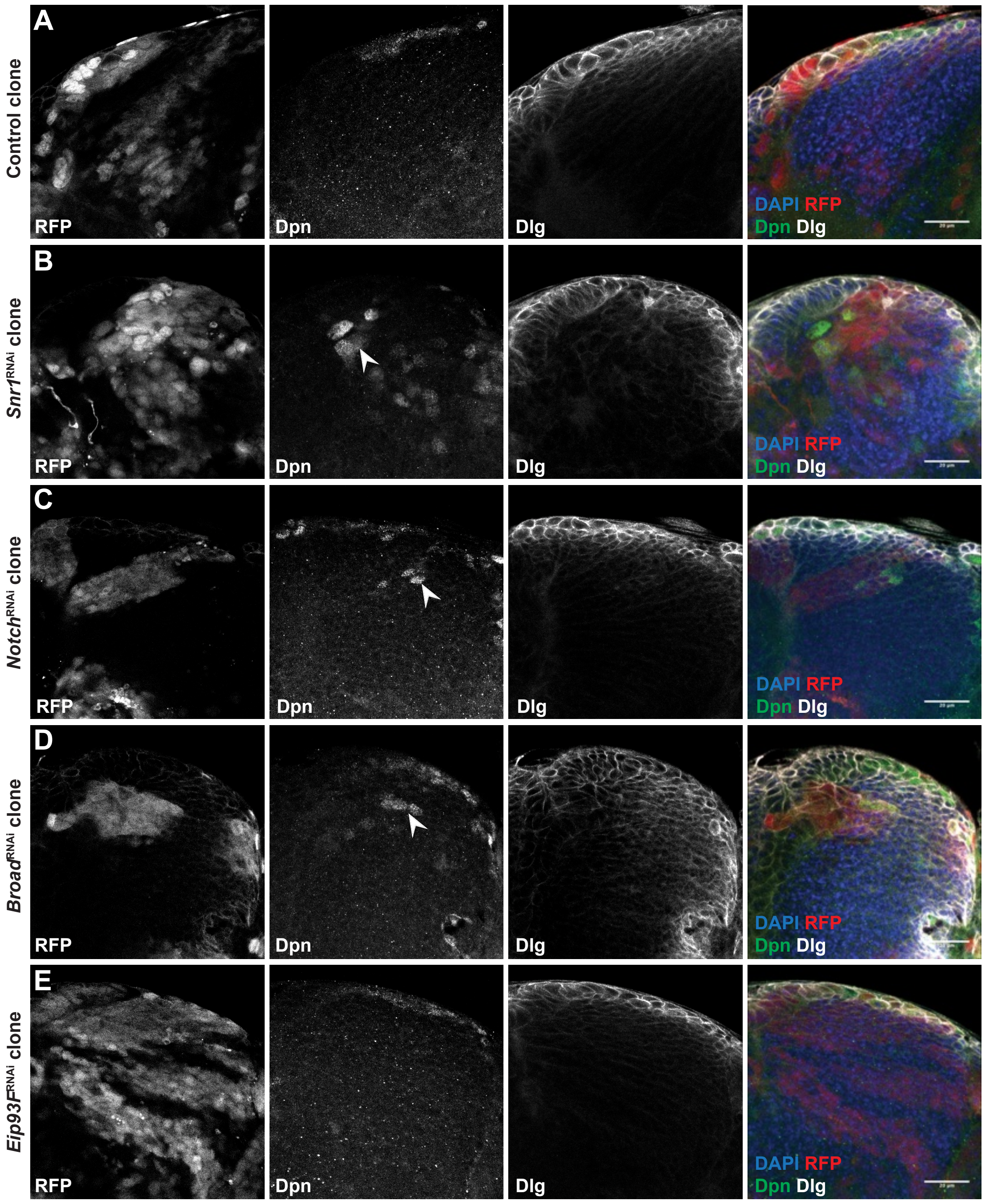

Fig. S8. Knockdown of Snr1 target genes recapitulate *Snr1*^{RNAi} phenotype. A) Control FLP-out clone shown in cross section. B) *Snr1*^{RNAi} clone. C) *Notch*^{RNAi} clone. B) *Broad*^{RNAi} clone. E) *Eip93F*^{RNAi} clone. A-E) Clones marked by RFP. Scale bars = 20 µm.

Development • Supplementary information

A

Gene	Average Log2 Fold Change	P-value
Eip93F	-1.120	5.82e-100
br	-0.996	2.90e-87
slp2	-0.979	4.97e-40
scro	-0.773	2.03e-23
SoxN	-0.543	2.57e-22
Vsx1	-0.550	2.46e-19
slp1	-0.497	8.65e-18
dmrt99B	-0.433	2.91e-13
hbn	-0.304	3.21e-09
opa	-0.458	4.46e-07
D	-0.389	9.46e-06

B

Gene	Average Log2 Fold Change	P-value
kn	-0.408	1.10e-279
hbn	-0.423	2.90e-265
Ets65A	-0.327	6.44e-251
D	-0.319	4.29e-218
Sox102F	-0.306	8.74e-190

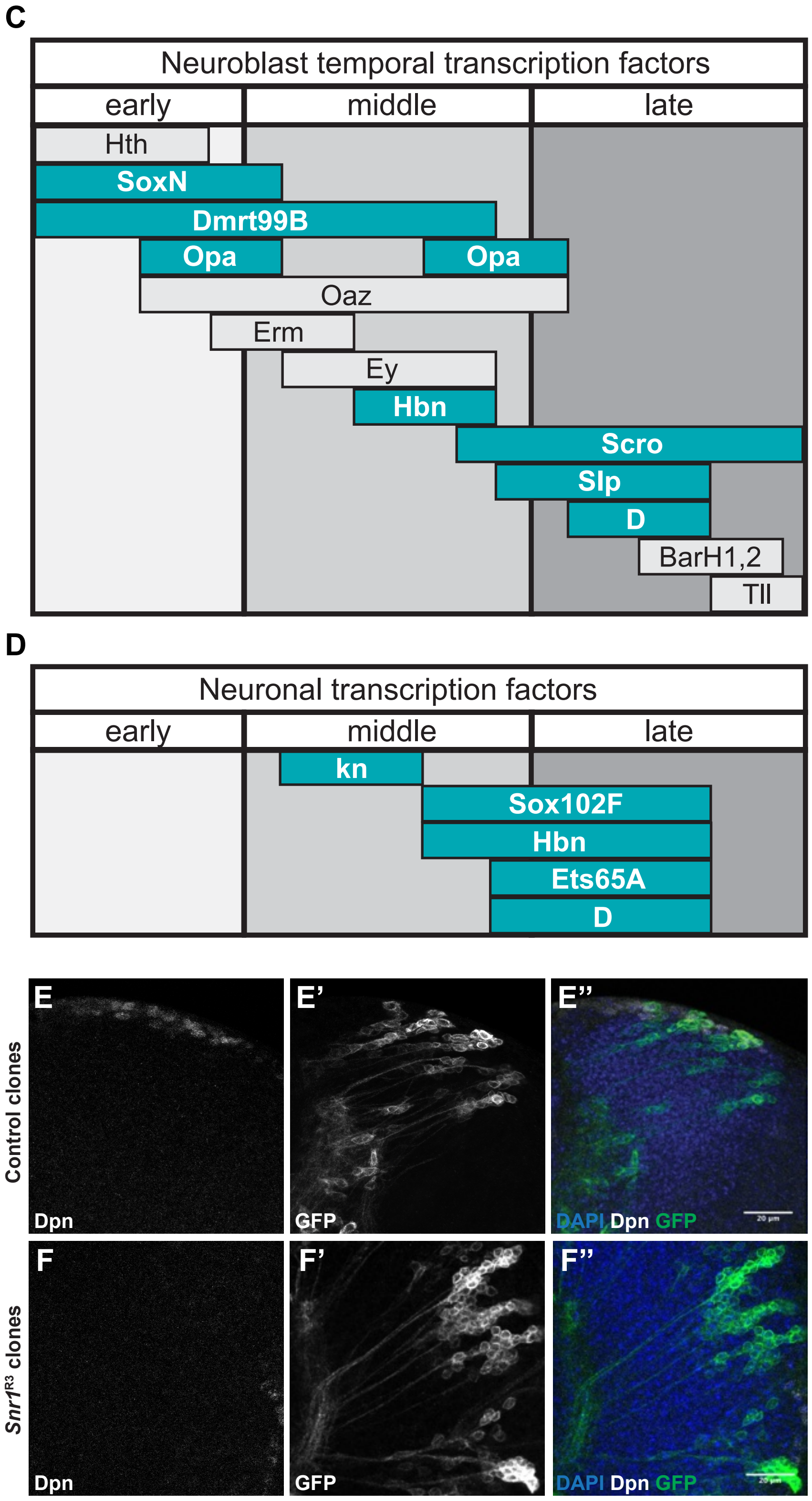

Fig. S9. Knockdown of Snr1 in neuroepithelial cells alters expression of temporal and spatial transcription factors in neuroblasts and neurons. A) Differential mRNA expression between control optic lobe neuroblast and optic lobe neuroblasts from brain with *Snr1*^{RNAi} in neuroepithelial cells. B) Differential expression between control optic lobe neurons and *Snr1* knockdown brains. C) Schematic of expression of temporal series of optic lobe neuroblast transcription factors. D) Subset of neuronal transcription factors which were differentially expressed in neurons in the *Snr1* knockdown in neuroepithelial cells. C and D were modified from Konstantinides et al. (2022) and Zhu et al. (2022). Differentially expressed genes shown in blue. E-E'') Control MARCM clones and F-F'') *Snr1*^{R3} MARCM clones generated in mid 3rd instar larvae. Scale bars = 20 µm.

Development • Supplementary information

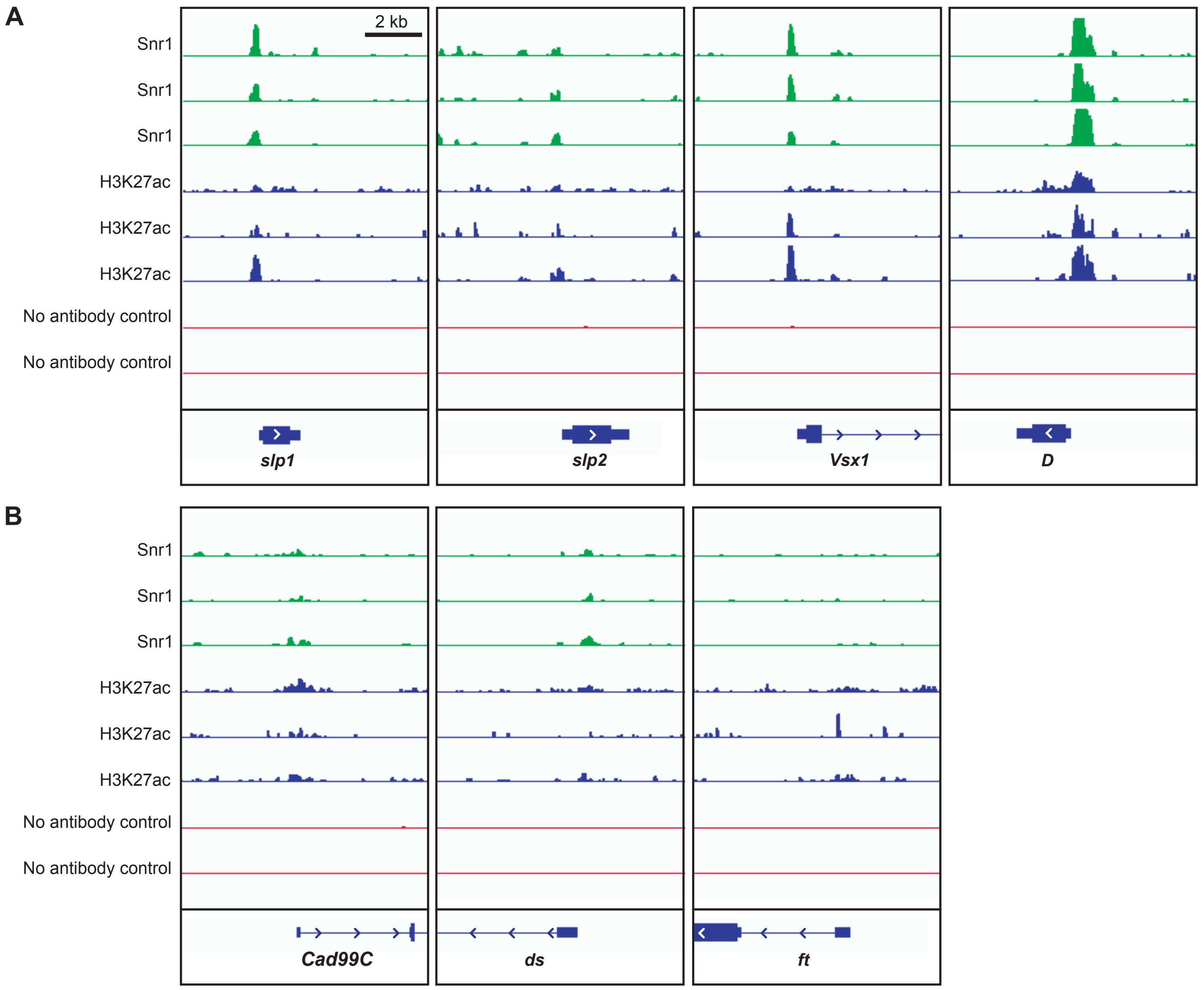

Fig. S10. Snr1 chromatin occupancy at genes involved in optic lobe development. Local chromatin landscapes at specific gene loci. A) Temporal and spatial transcription factors that had reduced expression due to Snr1 knockdown. B) Cadherin family members which increased expression due to *Snr1* knockdown.

Development • Supplementary information

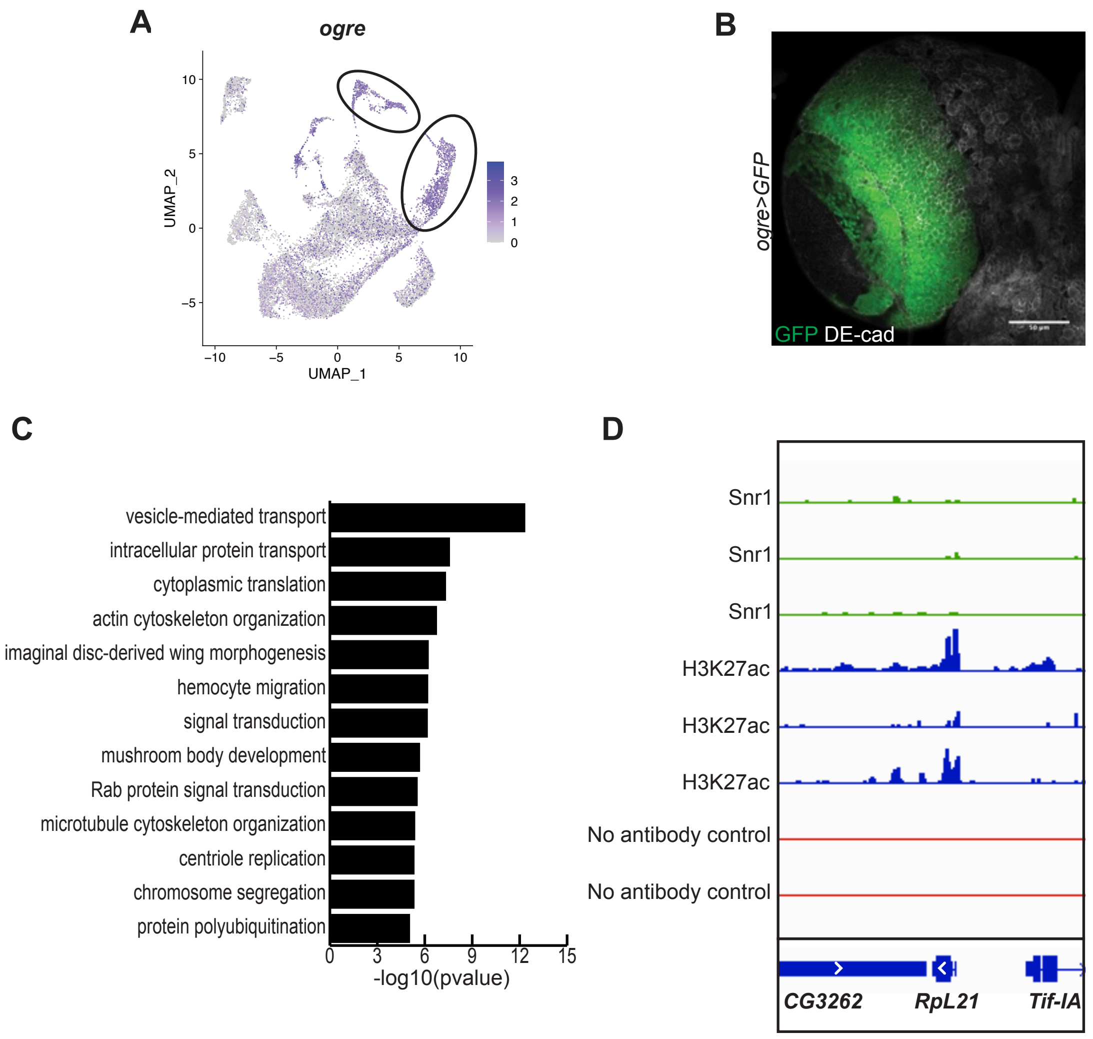

Fig. S11. Ogre is expressed in multiple cell types in the optic lobe. A) UMAP of the entire brain showing increased ogre expression in neuroepithelial and neuroblast clusters (circled). B) UAS-GFP expression under the control of the ogre-GAL4 promoter. C) Over-represented gene ontology terms associated with genes bound by H3K27ac but not Snr1. D) Example of gene (RpL21) with low Snr1 signal.

Table S1. List of differentially expressed genes between control and *Snr1*^{RNAi} neuroepithelial cell. Genes with p<0.01 shown.

Click here to download Table S1

Table S2. List of differentially expressed genes between control and *Snr1*^{RNAi} neuroblasts. Genes with p<0.01 shown.

Click here to download Table S2

Table S3. List of top 10% of genes with Snr1 occupancy in CUT&TAG analysis.

Click here to download Table S3

Konstantinides, N., Holguera, I., Rossi, A. M., Escobar, A., Dudragne, L., Chen, Y.-C., Tran, T. N., Martínez Jaimes, A. M., Özel, M. N., Simon, F. et al. (2022). A complete temporal transcription factor series in the fly visual system. *Nature* **604**, 316-322.

Zhu, H., Zhao, S. D., Ray, A., Zhang, Y. and Li, X. (2022). A comprehensive temporal patterning gene network in Drosophila medulla neuroblasts revealed by single-cell RNA sequencing. *Nature Communications* **13**, 1247.

Development • Supplementary information
