# Supplementary material for: A chromatin remodelling SWI/SNF subunit, Snr1, regulates neural stem cell determination and differentiation
Source: Development. 2023 Jun 30;150(13):dev201484. doi: 10.1242/dev.201484 (PMC10323235; doi:10.1242/dev.201484)
Supplement: Supplementary file 1 [file develop-150-201484_review_history.pdf]

A chromatin remodelling SWI/SNF subunit, Snr1, regulates neural stem cell determination and differentiation

Sophie E. Keegan, Julie Haskins, Andrew J. Simmonds and Sarah C. Hughes
DOI: 10.1242/dev.201484

Editor: Francois Guillemot

Review timeline

Original submission:	25 November 2022
Editorial decision:	23 January 2023
First revision received:	3 May 2023
Editorial decision:	17 May 2023
Second revision received:	26 May 2023
Accepted:	31 May 2023

Original submission

First decision letter

MS ID#: DEVELOP/2022/201484

MS TITLE: A chromatin remodelling SWI/SNF subunit Snr1 regulates neural stem cell determination and differentiation

AUTHORS: Sophie E. Keegan, Julie Haskins, Andrew J. Simmonds, and Sarah C. Hughes

I have now received the reports of three referees on your manuscript and I have reached a decision. The referees' comments are appended below, or you can access them online: please go to BenchPress and click on the 'Manuscripts with Decisions' queue in the Author Area.

As you will see, all the referees express great interest in your work, but they also have significant criticisms and recommend a substantial revision of your manuscript before we can consider publication. If you are able to revise the manuscript along the lines suggested, which may involve further experiments, I will be happy to receive a revised version of the manuscript. Your revised paper will be re-reviewed by one or more of the original referees, and acceptance of your manuscript will depend on your addressing satisfactorily the reviewers' major concerns. Please also note that Development will normally permit only one round of major revision. If it would be helpful, you are welcome to contact us to discuss your revision in greater detail. Please send us a point-by-point response indicating your plans for addressing the referee€™s comments, and we will look over this and provide further guidance.

Please attend to all of the reviewers' comments and ensure that you clearly highlight all changes made in the revised manuscript. Please avoid using 'Tracked changes' in Word files as these are lost in PDF conversion. I should be grateful if you would also provide a point-by-point response detailing how you have dealt with the points raised by the reviewers in the 'Response to Reviewers' box. If you do not agree with any of their criticisms or suggestions please explain clearly why this is so.

© 2023. Published by The Company of Biologists under the terms of the Creative Commons Attribution License (https://creativecommons.org/licenses/by/4.0/).

Reviewer 1

Advance summary and potential significance to field

The article "A chromatin remodelling SWI/SNF subunit Snr1 regulates neural stem cell determination and differentiation" aims to investigate the mechanism governing the formation of cellular diversity in the developing Drosophila Optic lobe. By employing conventional Drosophila genetic approaches, the authors showed that Snr1 regulates Optic lobe development, both at the level of neuroepithelium and neuroblasts. The authors suggested that the expression of Snr1 in the neuroepithelium is required for the maintenance of the neuroepithelial fate, and to prevent their precocious conversion into neuroblasts. Snr1 expression in the neuroblasts prolongs the expression of neuroblast markers whilst inhibiting their terminal differentiation. Via single-cell RNAseq, ectopic downregulation of snr1 in the neuroepithelium was shown to result in the downregulation of the Notch target E(spl)-C. In the neuroblasts, it caused the downregulation of the temporal factors Eip93f, Br and the spatial patterning gene vsx1. Concomitantly, CUT&Tag data suggested that Snr1 occupies the transcription start sites of E(spl)-C Eip93f, Br, temporal and spatial factors in the brain lobe. Altogether, the authors proposed that Snr1 present in the progenitor cells of the Optic lobe to maintain the chromatin accessibility of its target genes important for the specification of the neuroblasts from the neuroepithelium, and moreover neuroblast differentiation into neurons and glial cells.

Comments for the author

Major comments:
- Does Sn1R3 have a phenotype in other regions of the brain? The adult clones of Sn1R3 shown in Figure 4 are in the CB (not OL). Do the clones have a phenotype in other parts of the brain that has led to this phenotype?
- Do the RNAis which are used for the Sc RNAseq also produce the same phenotype in terms of persistence of NBs into adulthood. Or cause NE NB transition defects. This point is important, as the reagents used for sc RNAseq should really be able to recapitulate the mutant phenotype in order to decipher the mechanisms.
- The authors investigated why the Sn1R3 clones were smaller. They assayed ph3 and cell death and found neither were affected. So how is the clone smaller?
The authors should examine S phase of the cell cycle using EdU in clones, or look at cell cycle more carefully using FUCCI. It could be that M phase is not affected but S phase is.
- Following this point, the clones are smaller, but contain ectopic NBs this implies that each of the NBs must be proliferating slower in order to generate smaller clones, despite increased stem cell number. This point should be clarified.
- Figure 2: The authors stated that the twin spot clones are smaller than the wt, the size of the clone should be quantified, as should the number of ectopic NBs.
- Regarding Snr1 in the neuroepithelium, two possibilities should be considered: (1) Snr1 regulates neuroblast determination, independent of the proneural wave that progresses through the neuroepithelium to induce neuroblast formation, and (2) Snr1 regulates the timing of the proneural wave (either its initiation or rate of progression).
  o To show (1), loss of Snr1 in the neuroepithelium should be able to precociously produce neuroblast before the initiation of the proneural wave at early L3. For instance, c855a>snr1 RNAi or MARCM clones analysed in late L2 should have Dpn+ cells.
  o To show (2), loss of Snr1 in the neuroepithelium should be able to laterally shift the neuroepithelium/neuroblast border, either before (initiation) or after (rate of progression) the initiation of the wave.
- The conclusion "Snr1 is required to coordinate the transition from neuroepithelial cells to neuroblasts" is too vague.
  o Do Snr1-deificient clones have PatJ+Dpn+ double positive cells? If they do, Snr1 appears necessary for coordinating the transition from the neuroepithelial cell fate to the neuroblast fate. Otherwise, the use of "coordination" seems confusing.
  o An alternative conclusion can be "Snr1-deficient clones are extruded from the neuroepithelium and undergo precocious differentiation into neuroblast".

© 2023. Published by The Company of Biologists under the terms of the Creative Commons Attribution License (https://creativecommons.org/licenses/by/4.0/).

- The persistence of the NBs in Snr1 clones is interesting, however the authors do not investigate how these clones persist. Therefore, the mechanism is unclear. The authors can pursue this further by investigating whether the temporal series or the cell cycle is stalled, or whether the NE-NB transition delay underlies this phenotype. Temporal series could be implicated in this process, as lower levels of the temporal transcription factors were detected upon Snr1 knockdown.
- Notch mutant clones displayed similar phenotypes to snr1 mutant clones in this study. As such, to further show that Notch signalling is a target of Snr1, a rescue experiment should be done whereby Notch signalling is overexpressed in snr1 mutant clones.
- do changes in the expression of temporal and spatial transcription factors directly contribute to the altered organisation the optic lobe, as well as the persistence of the neuroblasts in adult animals? More functional test of these target genes should be done.
- The authors showed nicely that there are changes of Notch readouts as well as changes in the levels of Br and Eip93F. To show that these factors underlies some of the phenotypes caused by Sn1R3, genetic epistasis tests should be done.

Minor comments:

Methods
- scRNAseq: the author mentioned that ogre was used to subset Optic lobes cells. In the literature, ogre appears expressed only in the Optic lobe neuroepithelium. Thus, is this to re-cluster Optic lobe neuroepithelial cells only? If so, the method should specify as such.
- CUT&Tag: in the main text (excl. figure legends), the VNC was not abbreviated anywhere, so the author should use the full word here.
- S1, the driver used to perform the knockdown studies for Snr1 RNAi for westerns is not stated in the methods and figure legend.

Results
1. Snr1 regulates Optic lobe development
- Fig 1C: I suggest that the author may use other GAL4 drivers that are more general to the Optic lobe, such as hh-GAL4 (Erclik 2017) that is expressed in half of the Optic lobe instead of insc-GAL4 for Fig 1C. Additionally, this will provide a good internal control.
o For readers who are not familiar with the system, labels of the CNS regions (similar to Fig 1A) would be helpful in Fig 1C.
o In the text, the authors described the phenotypes observed in the Optic lobe as “altered organisation and shape, while other structures were similar to the control”. This is too general and should be more descriptive. E.g, if insc-GAL4 is used, can the size of the Optic lobe be quantified and compared between 2 conditions? What happens to the horseshoe shape of the proliferative centres?
- The expression pattern of Snr1 (Fig 2A) is mentioned in section 1 and thereby, is more appropriate to be in Fig 1 than Fig 2.
o It would be very helpful to label different cell types in Fig 2A like Fig 1B.
- In the sentence “We found relatively high levels of Snr1… surface of the brain (Fig 2A, Supp Fig 1A)”, citing Supp Fig 1A here adds no further information so should be omitted.
- Supp Fig 1C: it would be helpful to change the title to “Relative mRNA expression”, or adding a plot title “mRNA levels”
- 1K the authors stated that NBs were reduced, but rather I think the structure is disrupted and disorganised.

2. Neuroepithelial to neuroblast transition is disrupted in Snr1-deficient cells
- Because GAL4/UAS system was used in the previous section with cell type-specific GAL4s of both the neuroepithelium and the neuroblast, the authors should explain in the text why they use clonal induction in the following sections.
3. Snr1 deficient neuroblasts failed to differentiate
- Fig 3: RFP- clone is too small to clearly see any changes in Pros expression. The authors should provide another representative image.
- The 2nd paragraph of this section needs a clear conclusion about the effects on cell death and proliferation.
- Fig 4:

© 2023. Published by The Company of Biologists under the terms of the Creative Commons Attribution License (https://creativecommons.org/licenses/by/4.0/).

o Clones are in the Central brain, instead of the Optic lobe. The authors should provide another representative image that clearly shows clones in the Optic lobe with persistent neuroblasts.
o To show that Snr1-deficient neuroblasts do not differentiate, staining of neuronal markers like Elav for Snr1 mutant clones in the adult Optic lobe should be added.

4. Single cell RNA sequencing of the brain identified differentially expressed Snr1 targets
- The use of Ocho as a marker neuroepithelial cells needs citation.
- In the sentence "In wild type brains, E(spl)mgamma-GFP is expressed in neuroepithelial cells, suppressed in the transition zone and then expressed again in neuroblasts". The transition zone has not been introduced elsewhere and appeared a bit abruptly. Thus, it would be helpful to add a sentence about this in the introduction.
o The expression of E(spl)mgamma-GFP in the Optic lobe has also been shown by Contreras et al. (2018) with Dl marking the neuroepithelium. This should be cited.
- Fig 5F: the clone is small in the image. Moreover, at this late L3 stage the neuroepithelium remains only as a small proportion of the Optic lobe (most are Dpn+ at this stage). Therefore, it's difficult to assess how E(spl)mgamma expression changes in the neuroepithelial cells of the clone. To overcome this issue, an earlier timepoint should be imaged.
Fig 6A-D: the order of the genes shown are inconsistent with the legends.
- This section needs a clear conclusion about the differential expressed genes upon Snr1 loss of function.
5. Snr1 specifically targets brain development genes.
- This section needs a conclusion/suggestion for a model that recapitulates the findings of this study.

Data representations
- Boxplot was used throughout the article, which usually shows median lower, and upper interquartile. However, in the method, data = mean ± standard deviation so this is not actually shown in the plots? On that account, the authors should consider either changing the plot type or the legends.
- Some merge images lack labels. Although this is indicated in the legends it would be much better for the audience to follow with labels directly in the images.
- DCAD should be changed to DE-cadherin as a protein name.
Statistical analysis
- Student's t-test was used to quantify Supp Fig 2A which is not appropriate given that there are more than 2 samples to be compared. Hence, revisit statistical analysis should be revisited here.
Language and readability
- Overall, chosen language is simple to understand.
- Some sentences are unclear and need transitioning. E.g., "Somatic mosaic lineage tracing compared Snr1R3 cells to wild type cells generated in 'twin spot'
clones".
- The uses of "coordinate" and "maintain" appear too often with unclear meanings. Thus, depending on the context, the authors may need to reconsider word choices and extra explanations.

<u>Reviewer 2</u>

Advance summary and potential significance to field

In this manuscript, the authors investigate the role of Snr1 during Drosophila CNS development. Snr1 is a subunit of the SWI/SNF chromatin remodeling complex, and an ortholog of SMARCB1 in human whose inactivation causes rhabdoid tumors in children which are very aggressive, particularly when originating from the CNS. The precise function and role of Snr1/SMARCB1 during neurodevelopment remains poorly understood.

Here the authors describe loss of function phenotypes uncovered upon inactivation of Snr1 during optic lobe development. In addition, they identify deregulated target genes in various types of progenitors such as neuroepithelial cells and neuroblasts using single-cell RNA-seq and cut & tag.

© 2023. Published by The Company of Biologists under the terms of the Creative Commons Attribution License (https://creativecommons.org/licenses/by/4.0/).

This work has the potential to reveal significant and important insights into how Snr1 regulates temporal and other developmental transitions in neural progenitors. However, most data are rather superficially described and I have various concerns that need to be addressed for publications.

Comments for the author

Major points:

- The authors observed a disorganization of the optic lobe structure when Snr1 is knocked down either from neuroepithelial cells. This is apparently due to a reduction of the neuroepithelium size. However, there is no quantification of the phenotype. Please quantify neuroepithelium volume. The authors also mention that neuroblasts were reduced upon depletion of Snr1. However, this is not obvious at all from the Figure 1 M-O. Please quantify the volume of the optic lobe neuroblast compartment. This is also in contradiction with the later observation that Snr1 knockdown in neuroblasts causes neuroblast amplification. Please clarify.
- Figure 2A, the authors show an immunostaining performed with a new anti-Snr1 antibody that they have generated. In the optic lobe, the antibody labels Snr1 in the neuroepithelium and neuroblast layer that are at the surface of the CNS, but not the deeper neurons. This is surprising given that in the mammalian CNS, Snr1/SmarcB1 is supposed to be more or less ubiquitous. One possibility is that there is a penetration problem. Do you see the same restriction of Snr1 expression in the neuroblasts of the ventral nerve cord and central brain, or is it also expressed in the neurons and glia. In addition, this is also in contradiction with Figure 4I' where Snr1 is clearly expressed in neurons. Does it mean that immature neurons in larvae down-regulate Snr1 which is re-expressed then in mature neurons found in adults? Please clarify.
- The authors clearly identify a failure in medulla (optic lobe) neuroblast to properly differentiate upon Snr1 knockdown or in mutant clones (Fig.2C and 3A and B). In addition, they observe perdurance of ectopic neuroblasts in the adult CNS while neuroblast normally differentiate during metamorphosis. However, from Fig.4J, it is clear that ectopic adult NBs are not coming from the optic lobe as implied in the manuscript, but from the central brain. In fact, they likely come from type II NBs that have already been shown to induce NB tumors upon Snr1 depletion. This has been published in two papers from the Knoblich and Wang labs. http://dx.doi.org/10.1016/j.cell.2014.01.053 https://doi.org/10.7554/eLife.01906 It is unfortunate that these two papers are not cited in the manuscript as they are the two first studies that investigate Snr1 in the Drosophila CNS. These should absolutely be corrected. In addition, the authors should explicitly mention that ectopic neuroblasts in the adult CNS do not come from optic lobe neuroblasts and more clearly describe the fate of optic lobe neuroblasts lacking Snr1. Snr1 mutant clones appear absent from the optic lobe in Fig4. Does it mean that optic lobe cells lacking Snr1 are eliminated before adulthood?
- Fig S3: The authors use Tom, Ocho and Brd to identify neuroepithelial cells. Please add the reference indicating that these mRNAs characterize this cell type. They also say in the Mat&Met that they use Ogre to identify the neuroepithelial cells cluster. This is confusing. Please make it consistent throughout the manuscript.
- Fig5: It is not clear why the authors decided to concentrate on E(spl)mdelta and E(spl)m3 in Fig5C and D. Are they the most differentially expressed genes between neuroepithelial cells cluster 1 and cluster2?
- Then the authors decided to concentrate on E(spl)mgamma. Is this gene also differentially expressed between Cluster 1 and cluster 2 in the single-cell data ?
Please show. How differentially expressed is it also from the CTRL to KD condition?
Using a reporter gene, they show that E(spl)mgamma appear deregulated in the Snr1 KD clones spanning the neuroepithelium and neuroblasts. How consistent is it with single-cell data? In clones, E(spl)mgamma fails to be up-regulated in neuroblasts after the transition zone. Again, is it consistent with single-cell RNA-seq data?
- Does Snr1 continue to be down-regulated in NBs in the single-cell data when knocked down in the neuroepithelium only ?
- The authors should show a list of most differentially expressed genes in the neuroepithelium clusters of the control and KD conditions!
- Fig 6, please show umap with neuroblasts upon snr1 deregulation. As indicated in snr1-RNAi clones, Broad and Eip93F are also downregulated in the neuroepithelium. Is it consistent with single-cell data in Figure 5. In principle Broad and Eip93F should also appear as differentially expressed in the neuroepithelium between the control and KD conditions.

© 2023. Published by The Company of Biologists under the terms of the Creative Commons Attribution License (https://creativecommons.org/licenses/by/4.0/).

- Can your single-cell data be used to investigate whether lack of snr1 perturbs the differentiation and identity of optic lobe neurons?
- In the Cut & Tag experiments, it looks like snr1 binding always overlaps with active enhancers identified by H3K27ac. Is there any example of genes in which active enhancers have no Snr1 binding? What kind of genes are they?
- Interesting targets of Snr1 appear to be temporal transcription factors in medulla neuroblasts such as Slp1/2 and Dichaete. The authors refer to the 2013 Desplan lab paper. More recently, two papers have been published from the Desplan and Li labs that describe more exhaustively the temporal series in medulla neuroblasts. Did you look at the additional members of the series to see whether they are also regulated by Snr1? To validate this important data, the authors should look at the expression of these genes in Snr1 mutant clones. Are they completely repressed, or is their expression just delayed ?

Minor points : Fig 2 and 3 could be merged, such that additional figures describing in more details the identified snr1 targets in the neuroepithelium and neuroblasts could be added.

Reviewer 3

Advance summary and potential significance to field

In 'A chromatin remodelling SWI/SNF subunit Snr1 regulates neural stem cell determination and differentiation', Keegan et al. investigate the role of the chromatin remodelling protein Snr1 in Drosophila neuroblast development. They show that Snr1 is required for the correct timing of neuroblast specification from the neuroepithelium, and the subsequent differentiation of neuroblasts in the developing outer optic lobe. In snr1 mutants, neuroblasts form prematurely and additionally fail to down regulate neuroblast-specific markers. scRNA-seq analyses demonstrate that Snr1 regulates the expression of genes that are required for the neuroepithlial-neuroblast transition, including members of the Notch pathway. Their analysis also demonstrates that Snr1 is required in neuroblasts for the expression of genes required for neuroblast patterning, including the temporal transcription factors. Finally, the authors convincingly show that Snr1 occupies sites at the genomic loci associated with differentially expressed genes indicating that Snr1's role in regulating gene expression is direct.

This study will be of interest to researchers in both the chromatin remodelling and neurogenesis fields as the authors provide novel insights into the relationship between changes in chromatin accessibility and neural stem cell development. Of particular interest is the demonstration of a potentially direct link between Snr1, Notch signalling and the neuroepithelial to neuroblast transition. The scRNA-seq data will additionally provide a valuable resource for the optic lobe community. Several concerns (outlined below) should be addressed before the work is accepted for publication.

Comments for the author

1) In Figure 4 the authors show that snr1 RNAi clones persist in the adult as Dpn+ve cells. However, the cells shown in the figure are in the central brain (not the optic lobe) and are thus not neuroepithelium derived neuroblasts. The authors must show ectopic Dpn+ve cells in the adult medulla cortex if they make the argument that medulla neuroblasts persist into the adult in snr1 mutants.

2) The paper would be strengthened by a more detailed analysis of the snr1 neuroblast phenotypes. In snr1 mutant clones, cells are clustered in the larval medulla cortex where they maintain Dpn expression. Are these mutant cells trapped as neuroblasts that are unable to generate neurons? Including the neuronal marker ElaV in these stains would help to answer this question. To further determine the role of snr1 in neuroblasts, mutant clones should be generated in the mid-3rd instar to enrich for clones made in the neuroblast (and not the neuroepithelium).
In wild-type neuroblast-derived clones, a single chain of labeled cells will be visible with the parent neuroblast, GMCs and neurons labeled. It will be interesting to determine what these clones look like in snr1 mutants - for example, are multiple neuroblats labeled in a clone? Are neurons

© 2023. Published by The Company of Biologists under the terms of the Creative Commons Attribution License (https://creativecommons.org/licenses/by/4.0/).

generated in these clones? The insc>snr1 RNAi phenotype shown in Figure 1M-O should also be investigated further. Do the insc>snr1 RNAi medulla neuroblasts overproliferate?
The number of medulla neuroblasts in this background should be quantified and compared to wild-type.

3) With respect to the precocious neuroblast specification phenotype, a more detailed analysis would be helpful here to confirm that the ectopic neuroblasts observed in snr1 mutant clones are indeed due to a defect in the neuroepithelial-neuroblast transition. A lateral view of snr1 mutant clones in which the position of neuroblasts is visualized relative to the neuroepithelium and transition zone would better show if neuroblast formation is accelerated (this lateral view of clones has been shown in other papers that analyze the transition, such as Yasugi et al 2008, and indeed a similar view is shown in Figure 5E-F). Including the transition zone marker l(1)sc in these stains would also help show that the epithelial-neuroblast transition is sped up in snr1 clones.

4) The identification of Notch pathway genes as downstream targets of Snr1 is interesting and provides a potentially direct explanation for the precocious neuroblast formation phenotype observed in snr1 mutants. I would recommend further centering the results on this potential mechanism by including the CUT&Tag E(spl) data in the main figures. The model that Snr1 regulates the transition by promoting Notch signalling could be tested by attempting to rescue the snr1 precocious neuroblast phenotype by restoring Notch function with an E(spl) rescue construct.

Minor points:
1) The scRNA-seq analysis of optic lobe clusters should include a discussion of how the data compares to what was found in the analysis by Konstantinides et al.
2) The authors state that the central brain neuroblasts are unaffected in the insc>snr1 RNAi background. They should provide quantitative evidence that the number of central brain neuroblasts in unaffected in the knock-down.
3) In the Introduction it would be helpful to go into more detail into what role snr1 plays in the development of other Drosophila tissues.
4) In Figure 1, additional arrows and labels will help the reader understand the phenotypes. Additionally, the orientation of the optic lobe in Fig 1M-O should be the same as shown in the other panels.
5) The authors should state in the Results section that the role of snr1 is being investigated in the outer optic lobe epithelium (as opposed to the inner optic lobe epithelium, which gives rise to the lobula complex).

First revision

<u>Author response to reviewers' comments</u>

We thank the reviewers for their insightful comments and suggestions and do truly feel that these suggestions have improved the clarity and depth of analysis of our manuscript. We are pleased to report that we have addressed all comments from each reviewer. Please see our detailed responses to each reviewer below. The reviewer comments are included in italics. New data added to the revised version of the manuscript are referred to in bold text.

Overview of additional data for the revised manuscript: To address the reviewer comments we have added a total of 6 new figures (Figure 4, Figures S3, S7, S8, S9, S11) and 1 new Table (Table 1). The new Figures show the inappropriate perdurance of Dpn+ Snr1 loss of function clones in the adult optic lobe shown by Snr1R3 (Figure 3) and Snr1RNAi (Figure S3). We confirmed that Snr1 is regulating Notch signalling pathway using rescue experiments of pathway members (Figure S7), and epistasis analysis (Figure S8). We have also added new panels of data to 8 other figures. We have added 3 panels of quantification to Figure 1 (I, J, K) which includes that quantification of neuroepithelial volume, optic lobe neuroblasts and central brain neuroblasts. Figure 2 new panels F-G' which show early Snr1R3 clones in cross section. Figure 3 new panels C show abnormal expression of Elav in Snr1R3 clones. Figure 5 new panel E showing the differential expression of

© 2023. Published by The Company of Biologists under the terms of the Creative Commons Attribution License (https://creativecommons.org/licenses/by/4.0/).

E(spl) genes from the reanalysis of our scRNA-seq data. Figure 6 new panels A-D' early Snr1RNAi clones in cross section showing aberrant transition of neuroepithelial cells to neuroblasts. We have added data to supplemental Figure S2 panels E-F" Edu labelling in Snr1RNAi clones to address the smaller size of the clones, S4 panels B, C, new markers for neuroepithelial and neuroblast cells in scRNA-seq, and S6 panels A, C-L including identification of differentially expressed E(spl) genes and associated UMAPS.

Reviewer 1 Comments for the author
Major comments:
-Does Sn1R3 have a phenotype in other regions of the brain? The adult clones of Sn1R3 shown in Figure 4 are in the CB (not OL). Do the clones have a phenotype in other parts of the brain that has led to this phenotype?
We now show that Snr1R3 null clones induced in the optic lobe of larvae, are retained in the optic lobe of the adult brain and a portion of the cells continue to aberrantly express the stem cell neuroblast marker Dpn (Figure 4). As all neuroblasts of the optic lobe and central brain are eliminated at mid pupal stages (Homem et al., 2014, Maurange et al., 2008) it is significant that the clones of mutant Snr1 cells are retained in the adult brain optic lobe and express markers of stem cell-like features.
We do not observe significant effects in development in the rest of the larval brain when Snr1 is knocked down earlier in development. For example in Figure 1K we see only a slight increase in the number of neuroblasts in the central brain when Snr1 is reduced in all neuroblasts with insc-GAL4. Other studies have previously shown that Snr1 is in part required for proper progression of differentiation of type II neuroblast lineages in conjunction with other SWI/SNF complex member or HDAC3 (Koe et al., 2014; Eroglu et al., 2014). Thus, there may be other effects in the brain, but this is outside the focus of the current study.

-Do the RNAis which are used for the Sc RNAseq also produce the same phenotype in terms of persistence of NBs into adulthood. Or cause NE NB transition defects. This point is important, as the reagents used for sc RNAseq should really be able to recapitulate the mutant phenotype in order to decipher the mechanisms.
We have demonstrated that the Snr1RNAi line used for the scRNA-seq experiments produce the same phenotypes in the developing brain as the Snr1R3 null line. For example we show in Figure S3 that the Snr1RNAi FLP-out clones induced at the same point in development as the Snr1R3 clones (Figure 4) are retained into adulthood in the optic lobe and these clones continue to express Dpn in a proportion of the cells. In addition we show that use of the Snr1RNAi FLP-out clones in the larval brain produced clones that delaminate from the surface of the cortex and express Dpn deeper in the medulla region (Figure S7A, Figure S8B) which mimic the Snr1R3 clones (Figure 2A-B'). The Snr1RNAi clones imaged at the mid third instar stage (Figure 6D-D'') likewise show neuroepithelial cells prematurely converting to neuroblasts as seen in the Snr1R3 clones imaged at this stage, (Figure 2 F-G'''). In summary, we agree that this is an important consideration and we are confident that the use of the Snr1RNAi line recapitulates the Snr1 null clone phenotypes.

-The authors investigated why the Sn1R3 clones were smaller. They assayed ph3 and cell death and found neither were affected. So how is the clone smaller?
The authors should examine S phase of the cell cycle using EdU in clones, or look at cell cycle more carefully using FUCCI. It could be that M phase is not affected but S phase is.
This is an excellent point. We addressed this question using EdU labeling of larval brains and found that indeed the S phase of the cells in the Snr1RNAi clones is affected. There are fewer EdU positive cells present in the knock down clones as compared to controls (new Figure S2 F-F'' compared to E-E'', quantified in S2J) suggesting a slowing of the cell cycle which could account for the smaller clones size we observed.

-Following this point, the clones are smaller, but contain ectopic NBs, this implies that each of the NBs must be proliferating slower in order to generate smaller clones, despite increased stem cell number. This point should be clarified.

We have clarified this point in the text page 9 "Therefore the reduced size of the Snr1RNAi clones was likely due to a reduction in the rate of the cells entering the cell cycle which would suggest that neuroblasts are proliferating more slowly despite an increase in neuroblast numbers (Figure 3E)."

© 2023. Published by The Company of Biologists under the terms of the Creative Commons Attribution License (https://creativecommons.org/licenses/by/4.0/).

-Figure 2: The authors stated that the twin spot clones are smaller than the wt, the size of the clone should be quantified, as should the number of ectopic NBs.
Using Imaris, the clone volume and the number of Dpn positive cells have been quantified and this data is presented in Figure 3D and Figure 3E.

-Regarding Snr1 in the neuroepithelium, two possibilities should be considered: (1) Snr1 regulates neuroblast determination, independent of the proneural wave that progresses through the neuroepithelium to induce neuroblast formation, and (2) Snr1 regulates the timing of the proneural wave (either its initiation or rate of progression).
o To show (1), loss of Snr1 in the neuroepithelium should be able to precociously produce neuroblast before the initiation of the proneural wave at early L3. For instance, c855a>snr1 RNAi or MARCM clones analysed in late L2 should have Dpn+ cells.
o To show (2), loss of Snr1 in the neuroepithelium should be able to laterally shift the neuroepithelium/neuroblast border, either before (initiation) or after (rate of progression) the initiation of the wave.
-The conclusion “Snr1 is required to coordinate the transition from neuroepithelial cells to neuroblasts” is too vague.
We addressed these insightful possibilities using Snr1R3 and Snr1iRNA clones in early third instar larvae, when the majority of the cells are neuroepithelial and the transition zone is easily observable (Figure 2 F-G’’, Figure 6 D-D”). We show that Snr1 deficient neuroepithelial cells transition prematurely to neuroblasts ahead of the transition zone and delaminate from the epithelium suggesting that Snr1 is regulating the timing of the transition from neuroepithelial cells to neuroblasts. Further analysis of our scRNA-seq data shows that several Notch targets genes are reduced in the neuroepithelial cells as compared to control when Snr1 is reduced (Figure 6E). As loss of Notch signalling in the neuroepithelium triggers a premature transition to neuroblasts (Egger et al., 2010), this further supports our conclusion that Snr1 is regulating the timing of the transition between cell types.
We have strengthened these conclusions in the text of the manuscript.

oDo Snr1-deficient clones have PatJ+Dpn+ double positive cells? If they do, Snr1 appears necessary for coordinating the transition from the neuroepithelial cell fate to the neuroblast fate. Otherwise, the use of “coordination” seems confusing.
We do not observe PatJ+Dpn+ double positive cells in Snr1 null clones (e.g. Figure 2 D, D’), but rather cells in the clones express PatJ or Dpn or neither. Thus to avoid confusion we have removed the use of “coordination” throughout the text and state clearly that Snr1 is regulating specific target genes.

oAn alternative conclusion can be “Snr1-deficient clones are extruded from the neuroepithelium and undergo precocious differentiation into neuroblast”.
-The persistence of the NBs in Snr1 clones is interesting, however the authors do not investigate how these clones persist. Therefore, the mechanism is unclear. The authors can pursue this further by investigating whether the temporal series or the cell cycle is stalled, or whether the NE-NB transition delay underlies this phenotype. Temporal series could be implicated in this process, as lower levels of the temporal transcription factors were detected upon Snr1 knockdown.
We agree that neuroepithelial cells differentiate prematurely into neuroblasts. Via alteration of the timing of the dissection of Snr1 loss of function clones we observe clones that express neuroblast markers prematurely and that then delaminate from the surface of the optic lobe cortex and move more deeply into the brain (Figure 2 F-G’’, Figure 6 D-D”).
Our results also suggest that neuroblast development is altered when Snr1 expression is lost. We observe that Snr1R3 clones express neuroepithelial marker (PatJ; Figure 2D) and neural markers (Figure 3C) suggesting that differentiation of both neuroepithelial and neuroblasts are altered. We also see that the cell cycle is slowed in these clones (Figure S2E-F”) suggesting that this could contribute to the persistence of the clones. These data all support the role of Snr1 in regulation the determination and differentiation of neuroblasts. Additionally, the clones persist into adulthood (Figure 4, Figure S3) and continue to express neuroblast markers.
Alterations to the temporal series are likely implicated as our sequencing data does show reduced expression of a number of neuroblast temporal transcription factors (Figure S9A, C). Available antibodies for the different temporal factors to test this directly did not work in our hands. An in

© 2023. Published by The Company of Biologists under the terms of the Creative Commons Attribution License (https://creativecommons.org/licenses/by/4.0/).

depth analysis of the mechanism of the clone persistence is beyond the scope of the manuscript, but these would be interesting avenues of future research.

-Notch mutant clones displayed similar phenotypes to snr1 mutant clones in this study. As such, to further show that Notch signalling is a target of Snr1, a rescue experiment should be done whereby Notch signalling is overexpressed in snr1 mutant clones.
We have undertaken experiments testing the overexpression of Notch in Snr1RNAi clones and find that the overexpression of Notch is able to partially rescue the Snr1RNAi clones in that we observe that the clones are not delaminated from the optic lobe cortex, but do ectopically express Dpn in some of the mutant cells (Figure S7). We conclude that Notch signaling is a target of Snr1 and do not see full rescue potentially due the number of genes regulated by Snr1 at this stage that are contributing to the phenotype of the clones observed. We also conducted an epistasis experiment whereby FLP-out clones of NotchRNAi in neuroepithelial cells recapitulate the phenotype of Snr1 loss of function clones in that Dpn positive cells are observed deep in the medulla (Figure S8C). Together these results show that Notch signaling is a target of Snr1.

-do changes in the expression of temporal and spatial transcription factors directly contribute to the altered organisation the optic lobe, as well as the persistence of the neuroblasts in adult animals? More functional test of these target genes should be done.
As the effect of knocking down expression of many of the temporal and spatial transcription factors (including ey, slp1/2, D, vsx) have been published elsewhere (Li et al., 2013, Suzuki et al., 2013, Erclik et al., 2008), this was not repeated here. However, the effect of knocking down expression of Broad and Eip93F in the optic lobe has been less well characterized, so we performed additional experiments (as suggested in the next point) to test if these targets are contributing to the effects we see when Snr1 expression is reduced. These are late acting transcription factors and Eip93F has been shown by others to contribute to the persistence of neuroblast in adult animals (Pahl et al., 2019). While BroadRNAi did produce a similar phenotype to Snr1RNAi clones, Eip93FRNAi did not. We hypothesize that this is because reduced Snr1 expression would affect many target genes, and thus reduction of only one gene may not be sufficient to recapitulate the Snr1RNAi clone phenotype.

-The authors showed nicely that there are changes of Notch readouts as well as changes in the levels of Br and Eip93F. To show that these factors underlies some of the phenotypes caused by Sn1R3, genetic epistasis tests should be done.

We undertook a series of epistasis experiments whereby we generated knockdown of Notch, br or Eip93F using previously published RNAi lines (Kidd et al., 2015, Zhou et al., 2019, Pahl et al., 2019). We find that FLP-out clones of NotchRNAi and brRNAi recapitulated the phenotypes of Snr1RNAi clones with ectopic Dpn expressing cells that are found deeper in the medulla (Figure S8C, D). Interestingly Eip93FRNAi FLP-out clones appear more like control clones as discussed in the pervious point.

Minor comments:
Methods
-scRNAseq: the author mentioned that ogre was used to subset Optic lobes cells. In the literature, ogre appears expressed only in the Optic lobe neuroepithelium. Thus, is this to re-cluster Optic lobe neuroepithelial cells only? If so, the method should specify as such.
We have now included a new figure (Figure S11A) which shows the expression of ogre in both neuroepithelial cells and neuroblasts from our scRNA-seq (Figure S11A) and provide a representative image of UAS Stinger GFP expression under the control of ogre-GAL4 (Figure S11B) which shows that ogre is expressed throughout the optic lobe. This information is included in the Methods section on page 25 and 26.

-CUT&Tag: in the main text (excl. figure legends), the VNC was not abbreviated anywhere, so the author should use the full word here.
Ventral nerve cord is now defined in the results on page 5.
-S1, the driver used to perform the knockdown studies for Snr1 RNAi for westerns is not stated in the methods and figure legend.

© 2023. Published by The Company of Biologists under the terms of the Creative Commons Attribution License (https://creativecommons.org/licenses/by/4.0/).

The GAL4 line used for knockdown of Snr1 westerns was insc-GAL4 which is now included in the figure legend for Figure S1.

Results
1.Snr1 regulates Optic lobe development
-Fig 1C: I suggest that the author may use other GAL4 drivers that are more general to the Optic lobe, such as hh-GAL4 (Erclik 2017) that is expressed in half of the Optic lobe instead of insc-GAL4 for Fig 1C. Additionally, this will provide a good internal control.
We were not able to obtain this hh-GAL4, but have tested other GAL4 lines that express throughout the optic lobe (Optix-GAL4and Vsx-GAL4 Response to Reviewers Figure 1) and see similar effects to what is observed in Figure 1 (now Figure 1D). We include this as “data not shown” in the manuscript on page 5. This data provided the foundation to suggest that Snr1 was having an effect on optic lobe development which we then focus on and investigated more specifically using c855a-GAL4 and clonal analysis.

oFor readers who are not familiar with the system, labels of the CNS regions (similar to Fig 1A) would be helpful in Fig 1C.
The regions of the optic lobe we are focused on have now been labelled in Figure 1 C-D” and additionally the position of the transition zone has been marked in Figure 1B, E-E’’ and Figure 2F-G’” to make these key landmarks of the CNS more clear to all readers

oIn the text, the authors described the phenotypes observed in the Optic lobe as “altered organisation and shape, while other structures were similar to the control”. This is too general and should be more descriptive. E.g, if insc-GAL4 is used, can the size of the Optic lobe be quantified and compared between 2 conditions? What happens to the horseshoe shape of the proliferative centres?
We have marked where the optic lobe neuroepithelium and neuroblasts are present and have clarified our description in the text as “….resulted in mishapen optic lobe, while other structure were similar to control.” on page 5. We have outlined the optic lobes in Figure 1C and D to make this more obvious. We have also quantified the volume of the neuroepithelium in the c855a-GAL4 knockdown as compared to control (Figure 1I) and have found that there is a significant decrease in the neuroepithelium volume when Snr1 is knocked down.

-The expression pattern of Snr1 (Fig 2A) is mentioned in section 1 and thereby, is more appropriate to be in Fig 1 than Fig 2.
The panels showing expression pattern of Snr1 has been moved from Figure 2 and is now included as Figure 1E-E”, which also includes DE-cad and prospero expression.
oIt would be very helpful to label different cell types in Fig 2A like Fig 1B.
The transition zone and neuroepithelial and neuroblast cells are also now indicated in Figure 1 E-E’ as shown in Figure 1B.

-In the sentence “We found relatively high levels of Snr1… surface of the brain (Fig 2A, Supp Fig 1A)”, citing Supp Fig 1A here adds no further information so should be omitted.
We have removed the reference to Figure S1A in this sentence.

-Supp Fig 1C: it would be helpful to change the title to “Relative mRNA expression”, or adding a plot title “mRNA levels”
The title Snr1 mRNA expression has been added Figure S1C

-1K the authors stated that NBs were reduced, but rather I think the structure is disrupted and disorganised.
We agree that this description is more accurate and for Figure 1K (which is now Figure 1’) is now described as “reduced and disorganized” on page 6.

2.Neuroepithelial to neuroblast transition is disrupted in Snr1-deficient cells
-Because GAL4/UAS system was used in the previous section with cell type-specific GAL4s of both the neuroepithelium and the neuroblast, the authors should explain in the text why they use clonal induction in the following sections.

© 2023. Published by The Company of Biologists under the terms of the Creative Commons Attribution License (https://creativecommons.org/licenses/by/4.0/).

We have added in the description “This method was used to trace the development of Snr1 deficient cells in comparison to control cells in the same tissue” on page 7 to clarify why clonal analysis was used in addition to the UAS/GAL4 system.

3.Snr1 deficient neuroblasts failed to differentiate
-Fig 3: RFP- clone is too small to clearly see any changes in Pros expression. The authors should provide another representative image.

This image has been replaced with a representative image of Snr1R3 clones (Figure 3C) looking at the expression of the neuronal maker Elav. We find that clones are mixed, containing cells with premature and persistent Dpn signal and other cells in the clone expressing Elav. This suggests that cells in the loss of function Snr1 clones are not becoming neuroblasts at the correct time also resulting in differentiation defects.

-The 2nd paragraph of this section needs a clear conclusion about the effects on cell death and proliferation.

We have added the sentence “Therefore, the reduced size of Snr1RNAi clones was likely due to a reduction in the rate of cells entering the cell cycle” on page 9.

-Fig 4:
oClones are in the Central brain, instead of the Optic lobe. The authors should provide another representative image that clearly shows clones in the Optic lobe with persistent neuroblasts.

We have repeated these experiments and obtain clones in the optic lobe of adult brains (Figure 4). We have also done this with the Snr1RNAi FLP-out clones and observe a similar phenotype (Figure S3)

oTo show that Snr1-deficient neuroblasts do not differentiate, staining of neuronal markers like Elav for Snr1 mutant clones in the adult Optic lobe should be added.

We looked at the expression of the neuronal marker Elav in the larval Snr1R3 clones and found that at this stage there were already some cells that do express Elav, thus suggesting that in the cells in which Snr1 is lost, there is perturbation of differentiation at the correct time or location (Figure 3C).

4.Single cell RNA sequencing of the brain identified differentially expressed Snr1 targets
-The use of Ocho as a marker neuroepithelial cells needs citation.

The references for all of the neuroepithelial markers including Ocho and neuroblast markers are now included in the text on page 10 “(Brunet Avalos et al., 2019; Dillon et al., 2022; Egger et al., 2010; Konstantinides et al., 2022)”

-In the sentence “In wild type brains, E(spl)mgamma-GFP is expressed in neuroepithelial cells, suppressed in the transition zone and then expressed again in neuroblasts”. The transition zone has not been introduced elsewhere and appeared a bit abruptly. Thus, it would be helpful to add a sentence about this in the introduction.

We have added the sentence “Proneuronal genes are expressed in a region termed the transition zone (Figure 1B; arrowhead) that later converts neuroepithelial cells into neural stem cells (Egger et al., 2010; Yasugi et al., 2008).” to the introduction on page 3

oThe expression of E(spl)mgamma-GFP in the Optic lobe has also been shown by Contreras et al. (2018) with Dl marking the neuroepithelium. This should be cited.
This reference has been added on page 12.

-Fig 5F: the clone is small in the image. Moreover, at this late L3 stage, the neuroepithelium remains only as a small proportion of the Optic lobe (most are Dpn+ at this stage). Therefore, it’s

© 2023. Published by The Company of Biologists under the terms of the Creative Commons Attribution License (https://creativecommons.org/licenses/by/4.0/).

difficult to assess how E(spl)mgamma expression changes in the neuroepithelial cells of the clone.
To overcome this
issue, an earlier timepoint should be imaged.

We have extended the results using the E(spl)mgamma-GFP reporter and dissected the larvae containing Snr1RNAi clones at early third instar larval stage (Figure 6C-D") at which point the neuroepithelial cells comprise the majority of the optic lobe and we observed that the loss of function Snr1RNAi FLP-out clones have reduced E(spl)mgamma-GFP expression, prematurely delaminate from the surface of the optic lobe, and contain Dpn positive cells in front of the transition zone (Figure 6B, B' and D, D' as compared to Figure 6A, A' and C, C'). A similar effect is observed when the larval brains are dissected as later third instar (Figure 6F, F' as compared to Figure 6 E, E'). This shows that loss of Snr1 affects the timing of the transition of neuroepithelial cells into neuroblasts.

Fig 6A-D: the order of the genes shown are inconsistent with the legends.
-This section needs a clear conclusion about the differential expressed genes upon Snr1 loss of function.

Previous Fig 6A-D is now Figure 7 A-D and the order in which these genes are referred to in the text is changed.

5.Snr1 specifically targets brain development genes.
-This section needs a conclusion/suggestion for a model that recapitulates the findings of this study.

We have added in the sentence "Thus, Snr1 is associated with multiple genes required for the proper transition of neuroepithelial cells into neuroblasts and subsequent neuroblast differentiation " on page 15.

Data representations
-Boxplot was used throughout the article, which usually shows median, lower, and upper interquartile. However, in the method, data = mean ± standard deviation so this is not actually shown in the plots? On that account, the authors should consider either changing the plot type or the legends.
-Some merge images lack labels. Although this is indicated in the legends, it would be much better for the audience to follow with labels directly in the images.

-DCAD should be changed to DE-cadherin as a protein name.
The boxes in the box plots represents interquartile range (IQR) while the whiskers extend to nearest data point within 1.5 IQR of the box. This is now described in the methods.
Merged images now contain all labels of the antibodies used to provide clarity for the reader.
DCAD has been changed to DE-cadherin or DE-cad throughout the manuscript.

Statistical analysis
-Student's t-test was used to quantify Supp Fig 2A which is not appropriate given that there are more than 2 samples to be compared. Hence, revisit statistical analysis should be revisited here.
The statistical analysis throughout the manuscript have been checked and corrected as needed. We now used the one-way ANOVA with Tukey's post hoc test in Figure S2G-I as suggested. The statistics used are discussed in the Methods and Materials on page 26.

Language and readability
-Overall, chosen language is simple to understand.
-Some sentences are unclear and need transitioning. E.g., "Somatic mosaic lineage tracing compared Snr1R3 cells to wild type cells generated in 'twin spot' clones".
-The uses of "coordinate" and "maintain" appear too often with unclear meanings. Thus, depending on the context, the authors may need to reconsider word choices and extra explanations.

© 2023. Published by The Company of Biologists under the terms of the Creative Commons Attribution License (https://creativecommons.org/licenses/by/4.0/).

We have carefully edited the manuscript and ensured that the description of the types of clones are clear. We have also removed many of the descriptors of coordinate and maintain to ensure clarity of the description of our results.

Reviewer 2 Comments for the author
Major points:
-The authors observed a disorganization of the optic lobe structure when Snr1 is knocked down either from neuroepithelial cells. This is apparently due to a reduction of the neuroepithelium size. However, there is no quantification of the phenotype. Please quantify neuroepithelium volume. The authors also mention that neuroblasts were reduced upon depletion of Snr1. However, this is not obvious at all from the Figure 1 M-O. Please quantify the volume of the optic lobe neuroblast compartment. This is also in contradiction with the later observation that Snr1 knockdown in neuroblasts causes neuroblast amplification. Please clarify.
We have quantified the neuroepithelial volume when Snr1 is reduced using c855a-GAL4 (Figure 1I) and we find that the volume is significantly reduced in the Snr1 knock down cells supporting the observation that there is a decrease in the size of the lobe neuroepithelium in comparison to the control.
In addition, we have quantified the number of optic lobe neuroblasts when insc-GAL4 is used to reduce Snr1 expression and there is actually a decrease in the number of neuroblasts (Figure 1J), which we have adjusted in our description of the results. In addition we also quantified the number of neuroblasts in the central brain as insc-GAL4 is expressed in all neuroblasts and observation that there is a slight increase in number when Snr1 is reduced (Figure 1K). So while the primary effect of loss of Snr1 is in the optic lobe, which is what this manuscript focuses on, there may be subtle effects in the central brain but investigation of these effects is beyond the scope of the manuscript.

-Figure 2A, the authors show an immunostaining performed with a new anti-Snr1 antibody that they have generated. In the optic lobe, the antibody labels Snr1 in the neuroepithelium and neuroblast layer that are at the surface of the CNS, but not the deeper neurons. This is surprising given that in the mammalian CNS, Snr1/SmarcB1 is supposed to be more or less ubiquitous. One possibility is that there is a penetration problem. Do you see the same restriction of Snr1 expression in the neuroblasts of the ventral nerve cord and central brain, or is it also expressed in the neurons and glia. In addition, this is also in contradiction with Figure 4I' where Snr1 is clearly expressed in neurons. Does it mean that immature neurons in larvae down-regulate Snr1 which is re-expressed then in mature neurons found in adults? Please clarify.

We agree with this point and have adjusted how we discuss the protein expression as observed in Figure 1E'. As there is Snr1 expression in the adult brains, in presumptive neurons in the non-clonal regions, it is possible that there is an issue with antibody penetration into the deeper medulla.

-The authors clearly identify a failure in medulla (optic lobe) neuroblast to properly differentiate upon Snr1 knockdown or in mutant clones (Fig.2C and 3A and B). In addition, they observe perdurance of ectopic neuroblasts in the adult CNS while neuroblast normally differentiate during metamorphosis. However, from Fig.4J, it is clear that ectopic adult NBs are not coming from the optic lobe as implied in the manuscript, but from the central brain. In fact, they likely come from type II NBs that have already been shown to induce NB tumors upon Snr1 depletion. This has been published in two papers from the Knoblich and Wang labs.
http://dx.doi.org/10.1016/j.cell.2014.01.053
https://doi.org/10.7554/eLife.01906
It is unfortunate that these two papers are not cited in the manuscript as they are the two first studies that investigate Snr1 in the Drosophila CNS. These should absolutely be corrected. In addition, the authors should explicitly mention that ectopic neuroblasts in the adult CNS do not come from optic lobe neuroblasts and more clearly describe the fate of optic lobe neuroblasts lacking Snr1. Snr1 mutant clones appear absent from the optic lobe in Fig4. Does it mean that optic lobe cells lacking Snr1 are eliminated before adulthood?
As discussed above with Reviewer 1 comments we have repeated generating clones in the neuroepithelium and consistently obtain clones in the optic lobe of the adult brain. As all neuroblasts of the optic lobe and central brain are eliminated at mid pupal stages (Homem et al 2014, Murange et al 2008) it is significant that the clones of mutant Snr1 cells are retained in the adult brain optic lobe and express contain Dpn expressing cells. As our focus is on the development

© 2023. Published by The Company of Biologists under the terms of the Creative Commons Attribution License (https://creativecommons.org/licenses/by/4.0/).

of the optic lobe we did not further evaluate clones that might be derived from the central brain and have replaced these images.

-Fig S3: The authors use Tom, Ocho and Brd to identify neuroepithelial cells. Please add the reference indicating that these mRNAs characterize this cell type. They also say in the Mat&Met that they use Ogre to identify the neuroepithelial cells cluster. This is confusing. Please make it consistent throughout the manuscript.
The references for all of the neuroepithelial markers including Ocho, Brd and neuroblast markers are now included in the text on page 10 "(Brunet Avalos et al., 2019; Dillon et al., 2022; Egger et al., 2010; Konstantinides et al., 2022)".

-Fig5: It is not clear why the authors decided to concentrate on E(spl)mdelta and E(spl)m3 in Fig5C and D. Are they the most differentially expressed genes between neuroepithelial cells cluster 1 and cluster2?
Several members of the E(spl) gene complex had high relative expression in neuroepithelial cells cluster 1 (Enhancer of split Complex (E(spl)-C) [(E(spl)m4-BFM, log2FC=3.63; E(spl)malpha-BFM, log2FC=3.33; E(spl)mdelta-HLH, log2FC=3.09; E(spl)m3-HLH, log2FC=2.56; E(spl)mgamma-HLH, log2FC=2.44, p<1e-100]) listed on page 11 which is why these were chosen to further investigate the potential mechanism of the effect of the loss of Snr1 in neuroepithelial. The UMAPs in Figure 5C and D have been replaced with the two E(spl) genes with the highest differential expression between control and Snr1RNAi cells to correspond with the data shown in Figure 5E.

-Then the authors decided to concentrate on E(spl)mgamma. Is this gene also differentially expressed between Cluster 1 and cluster 2 in the single-cell data ? Please show. How differentially expressed is it also from the CTRL to KD condition?
Using a reporter gene, they show that E(spl)mgamma appear deregulated in the Snr1 KD clones spanning the neuroepithelium and neuroblasts. How consistent is it with single-cell data? In clones, E(spl)mgamma fails to be up-regulated in neuroblasts after the transition zone. Again, is it consistent with single-cell RNA-seq data?
A UMAP of E(spl)mgamma expression is now shown in Figure 5C and the differential expression between control and Snr1RNAi cells is shown in Figure 5E and Figure S6A. We now also show UMAPs of E(spl)mgamma expression separately in control (Figure S6E) or Snr1RNAi cells (Figure S6J) which show expression of E(spl)mgamma in neuroepithelial cells and neuroblasts.

-Does Snr1 continue to be down-regulated in NBs in the single-cell data,
when knocked down in the neuroepithelium only?
We have included UMAPs of Snr1 expression separately in control (Figure S6D) or Snr1RNAi optic lobe cells (Figure S6I) which show that Snr1 continues to be down-regulated in neuroblasts following knockdown in neuroepithelial cells.

-The authors should show a list of most differentially expressed genes in the neuroepithelium clusters of the control and KD conditions!
This has been included as Table 1.

-Fig 6, please show umap with neuroblasts upon snr1 deregulation. As indicated in snr1-RNAi clones, Broad and Eip93F are also downregulated in the neuroepithelium. Is it consistent with single-cell data in Figure 5. In principle, Broad and Eip93F should also appear as differentially expressed in the neuroepithelium between the control and KD conditions.
This has now been included in Figure S6 (Figure S6F, G, K, L) showing that Broad and Eip93F are differentially expressed in the neuroepithelium upon Snr1 knockdown.

-Can your single-cell data be used to investigate whether lack of snr1 perturbs the differentiation and identity of optic lobe neurons?
We have used our scRNA-seq data to look for differential expression of the known neuronal transcription factors identified in the recent Desplan lab papers. We observe that the middle and late acting transcription factors kn, Sox102F, Ets65A and D are differentially expressed when Snr1 is knocked down in the neuroepithelial cells (Figure S9A, C). Thus, loss of Snr1 does perturb the differentiation and identity of optic lobe neurons and this data will provide the basis of future experiments to analyze how neuronal identity is being determined.

© 2023. Published by The Company of Biologists under the terms of the Creative Commons Attribution License (https://creativecommons.org/licenses/by/4.0/).

-In the Cut&Tag experiments, it looks like snr1 binding always overlaps with active enhancers identified by H3K27ac. Is there any example of genes in which active enhancers have no Snr1 binding? What kind of genes are they?
We have curated a list of gens that have active enhancers (H3K27ac) but do not have Snr1 binding and interestingly there are very few of these genes in our dataset from the whole brain. The classes of genes identified primarily contain housekeeping and mitotic genes. We have included this new analysis in Figure S11C and D.
What this result suggests is that Snr1 is required specifically in the timing and differentiation of genes that function in patterning the developing brain as shown in Figure 8B in our ORA analysis.

-Interesting targets of Snr1 appear to be temporal transcription factors in medulla neuroblasts such as Slp1/2 and Dichaete. The authors refer to the 2013 Desplan lab paper. More recently, two papers have been published from the Desplan and Li labs that describe more exhaustively the temporal series in medulla neuroblasts. Did you look at the additional members of the series to see whether they are also regulated by Snr1? To validate this important data, the authors should look at the expression of these genes in Snr1 mutant clones. Are they completely repressed, or is their expression just delayed ?

We attempted to look at temporal factors with available antibodies (e.g. Hth, eyeless) but they did not work in our hands. Thus, we have reanalyzed our scRNA-seq data to look at the expanded temporal series and find that a majority are differentially expressed when Snr1 is knocked down in neuroepithelial cells (c558a-GAL4) which are listed in Figure S9A and schematically shown in Figures S9C. We observe that their expression is reduced. Additionally, we note that neuronal transcription factors from the same dataset when Snr1 is reduced in neuroepithelial cells are also effected in the middle and late stages of the temporal series (Figure S9b, D). We find that the expression of these neural transcription factors are reduced (Figure S9B).

Minor points : Fig 2 and 3 could be merged, such that additional figures describing in more details the identified snr1 targets in the neuroepithelium and neuroblasts could be added.
We have rearranged data in many of the figures while maintaining a sufficient size for each panel for ease of reading. We have added new data to many of the original figure and have added more supplementary figures.

Reviewer 3 Comments for the author
1) In Figure 4 the authors show that snr1 RNAi clones persist in the adult as Dpn+ve cells. However, the cells shown in the figure are in the central brain (not the optic lobe) and are thus not neuroepithelium derived neuroblasts. The authors must show ectopic Dpn+ve cells in the adult medulla cortex if they make the argument that medulla neuroblasts persist into the adult in snr1 mutants.

As discussed above with Reviewer 1 and Reviewer 2, we repeated the experiments multiple times and consistently show that either Snr1R3 null clones induced in the optic lobe of larvae, are retained in the optic lobe of the adult brain and a portion of the cells continue to aberrantly express the stem cell neuroblast marker Dpn (Figure 4). We have repeated this analysis to show that the loss of Snr1 by Snr1RNAi FLP-out clones (Figure S3) has the same effect of persistent clones which aberrantly express Dpn.

2) The paper would be strengthened by a more detailed analysis of the snr1 neuroblast phenotypes. In snr1 mutant clones, cells are clustered in the larval medulla cortex where they maintain Dpn expression. Are these mutant cells trapped as neuroblasts that are unable to generate neurons? Including the neuronal marker ElaV in these stains would help to answer this question.
We generated Snr1R3 clones early in larval development and found that some cells within the clone express Elav, thus the aberrant neuroblasts in the Snr1 null clones are able to generate neurons (Figure 3C), albeit in a disrupted pattern with some cells appearing to have brighter fluorescence than other cells. This suggests that the loss of Snr1 results in cells that are dysregulated in terms of when they become neuroblast and differentiate into neurons.
To further determine the role of snr1 in neuroblasts, mutant clones should be generated in the mid-3rd

© 2023. Published by The Company of Biologists under the terms of the Creative Commons Attribution License (https://creativecommons.org/licenses/by/4.0/).

instar to enrich for clones made in the neuroblast (and not the neuroepithelium). In wild-type neuroblast-derived clones, a single chain of labeled cells will be visible with the parent neuroblast, GMCs and neurons labeled. It will be interesting to determine what these clones look like in snr1 mutants - for example, are multiple neuroblats labeled in a clone? Are neurons generated in these clones? The insc>snr1 RNAi phenotype shown in Figure 1M-O should also be investigated further. Do the insc>snr1 RNAi medulla neuroblasts overproliferate? The number of medulla neuroblasts in this background should be quantified and compared to wild-type.
As suggested, we generated Snr1R3 clones at the mid-3rd instar stage to capture the transition from the neuroblasts into neurons (Figure S9E-F’’). These brains were imaged in the lateral view which show in the Snr1 null cells that the morphology is altered and cells appear clumped closer to the surface as compared to the wild type clones in which the columnar organization of the cells is observed as previously reported by others. We did not see perdurance of the Dpn marker or multiple Dpn cells in clones generated at this stage (Figure S9E, F). For the inscGal4;snr1 RNAi phenotype, the number of medulla neuroblasts was quantified and is now included as Figure 1J.

3) With respect to the precocious neuroblast specification phenotype, a more detailed analysis would be helpful here to confirm that the ectopic neuroblasts observed in snr1 mutant clones are indeed due to a defect in the neuroepithelial-neuroblast transition. A lateral view of snr1 mutant clones in which the position of neuroblasts is visualized relative to the neuroepithelium and transition zone would better show if neuroblast formation is accelerated (this lateral view of clones has been shown in other papers that analyze the transition, such as Yasugi et al 2008, and indeed a similar view is shown in Figure 5E-F). Including the transition zone marker l(1)sc in these stains would also help show that the epithelial-neuroblast transition is sped up in snr1 clones.
As discussed in the comments from Reviewer 1 we addressed these insightful possibilities using Snr1R3 and Snr1RNAi clones in early third instar larvae, when the majority of the cells are neuroepithelial and the transition zone is easily observable (Figure 2 F-G’’, Figure 6 D-E”). We now show that Snr1 deficient neuroepithelial cells transition prematurely to neuroblasts ahead of the transition zone and delaminate from the epithelium suggesting that Snr1 is regulating the timing of the transition from neuroepithelial cells to neuroblasts. Most clones were images in cross-section because the clones were extruded from the neuroepithelium and were located deep inside the brain. Clones could not easily be visualized from the lateral view because they were partially located below the brain surface (an example of this is the lower clone in Figure 2E). The transition zones have been marked by the expression of PatJ in Figure 2 and the expression of the E(spl)gamma in Figure 6. L(1)sc antibody in hand did not robustly work in our hands

4) The identification of Notch pathway genes as downstream targets of Snr1 is interesting and provides a potentially direct explanation for the precocious neuroblast formation phenotype observed in snr1 mutants. I would recommend further centering the results on this potential mechanism by including the CUT&Tag E(spl) data in the main figures. The model that Snr1 regulates the transition by promoting Notch signalling could be tested by attempting to rescue the snr1 precocious neuroblast phenotype by restoring Notch function with an E(spl) rescue construct.

We have moved the CUT&TAG analysis into Figure 8E to more strongly support the role of Snr1 targeting Notch signaling. We also used Notch and two E(spl) targets to attempt to rescue the Snr1RNAi clones phenotypes. We found that overexpression of Notch and E(spl)m4 partially rescues this phenotype while E(spl)m7 does not. This is included as Figure S7. This data is interesting in that it reinforces Notch signalling as a target of Snr1 but the lack of a full rescue highlights the importance of precise regulation of this pathway in the optic lobe.

Minor points:

1) The scRNA-seq analysis of optic lobe clusters should include a discussion of how the data compares to what was found in the analysis by Konstantinides et al.

We have used the analysis by Konstantinides et al to analyze our scRNA-seq data and for analysis of changes in the temporal neuroblast markers and neural transcription factors. This is included in the results section pages 10 and 11.

© 2023. Published by The Company of Biologists under the terms of the Creative Commons Attribution License (https://creativecommons.org/licenses/by/4.0/).

2) The authors state that the central brain neuroblasts are unaffected in the inscGal4;snr1 RNAi background. They should provide quantitative evidence that the number of central brain neuroblasts in unaffected in the knock-down.

We have quantified the number of neuroblasts in the central brain (Figure 1K) and observe a slight increase in the number. There may be subtle effects in the central brain which was outside of the focus of our manuscript.

3) In the Introduction it would be helpful to go into more detail into what role snr1 plays in the development of other Drosophila tissues.

This information has been expanded upon to provide context to the role of Snr1 in development in general in Drosophila on page 4 and 5.
4) In Figure 1, additional arrows and labels will help the reader understand the phenotypes. Additionally, the orientation of the optic lobe in Fig 1M-O should be the same as shown in the other panels

We have added additional arrows to mark key points of the optic lobe that we are discussing and the transition zone in Figure 1B, C', D', E-E" as well as Figure 3F-G". The brain shown in Figure 1M-O, now H-H'' is disorganized because of the knockdown but is in the same orientation as the other panels.

5) The authors should state in the Results section that the role of snr1 is being investigated in the outer optic lobe epithelium (as opposed to the inner optic lobe epithelium, which gives rise to the lobula complex).

This oversight has been corrected on page 6

References for Response to Reviewers

Egger, B., Gold, K. S. and Brand, A. H. (2010). Notch regulates the switch from symmetric to asymmetric neural stem cell division in the Drosophila optic lobe. Development, 137(18), 2981-2987.
Erclik, T., Hartenstein, V., Lipshitz, H. D., & McInnes, R. R. (2008). Conserved role of the Vsx genes supports a monophyletic origin for bilaterian visual systems. Current Biology, 18(17), 1278-1287.
Eroglu, E., Burkard, T. R., Jiang, Y., Saini, N., Homem, C. C., Reichert, H., & Knoblich, J. A. (2014). SWI/SNF complex prevents lineage reversion and induces temporal patterning in neural stem cells. Cell, 156(6), 1259-1273.
Homem, C. C., Steinmann, V., Burkard, T. R., Jais, A., Esterbauer, H., & Knoblich, J. A. (2014). Ecdysone and mediator change energy metabolism to terminate proliferation in Drosophila neural stem cells. Cell, 158(4), 874-888.
Kidd, S., Struhl, G., & Lieber, T. (2015). Notch is required in adult Drosophila sensory neurons for morphological and functional plasticity of the olfactory circuit. PLoS genetics, 11(5), e1005244.
Koe, C. T., Li, S., Rossi, F., Wong, J. J. L., Wang, Y., Zhang, Z., ... & Wang, H. (2014). The Brm-HDAC3-Erm repressor complex suppresses dedifferentiation in Drosophila type II neuroblast lineages. Elife, 3, e01906.
Li, X., Erclik, T., Bertet, C., Chen, Z., Voutev, R., Venkatesh, S., ... and Desplan, C. (2013). Temporal patterning of Drosophila medulla neuroblasts controls neural fates. Nature, 498(7455), 456-462.
Maurange, C., Cheng, L., & Gould, A. P. (2008). Temporal transcription factors and their targets schedule the end of neural proliferation in Drosophila. Cell, 133(5), 891-902.
Pahl, M. C., Doyle, S. E., & Siegrist, S. E. (2019). E93 integrates neuroblast intrinsic state with developmental time to terminate MB neurogenesis via autophagy. Current Biology, 29(5), 750-762.
Suzuki, T., Kaido, M., Takayama, R., & Sato, M. (2013). A temporal mechanism that produces neuronal diversity in the Drosophila visual center. Developmental biology, 380(1), 12-24.
Zhou, Y., Yang, Y., Huang, Y., Wang, H., Wang, S., & Luo, H. (2019). Broad promotes neuroepithelial stem cell differentiation in the Drosophila optic lobe. Genetics, 213(3), 941-951.

Figure 1 for Response to reviewers uploaded separately

© 2023. Published by The Company of Biologists under the terms of the Creative Commons Attribution License (https://creativecommons.org/licenses/by/4.0/).

Second decision letter

MS ID#: DEVELOP/2022/201484

MS TITLE: A chromatin remodelling SWI/SNF subunit Snr1 regulates neural stem cell determination and differentiation

AUTHORS: Sophie E Keegan, Julie Haskins, Andrew J Simmonds, and Sarah C Hughes

I have now received the reports of two of the referees who reviewed the earlier version of your manuscript, and have reached a decision. The referees' comments are appended below, or you can access them online: please go to BenchPress and click on the 'Manuscripts with Decisions' queue in the Author Area.

The reviewers' overall evaluation is positive and we would like to publish a revised manuscript in Development, provided that the remaining referees' comments can be satisfactorily addressed. Please attend to all of the reviewers' comments in your revised manuscript and detail them in your point-by-point response. If you do not agree with any of their criticisms or suggestions explain clearly why this is so.

Reviewer 1

Advance summary and potential significance to field

see previous review comments

Comments for the author

- The authors have addressed most of my concerns. There are a few more modifications that can be made in order to improve the manuscript.
-
1. Regarding the epistatic tests of Snr1 with Br and Eip93F requested, the authors have only done Br and Eip93F knockdown by RNAi, which phenocopied Snr1RNAi. They have not done epistasis of Br or Eip93F and Snr1. I suggest they change the wording where they say they have done the epistasis, and just say that Br and Eip93F knockdown phenocopied Snr1 RNAi.
2. Description of optic lobe development needs to be a bit more comprehensive. In the result 1, the idea of “outer neuroepithelium” appeared quite abruptly, which should be first mentioned in the introduction. Inspirations could be found in the review of Apitz and Salecker, 2014.
3. In Figure 1, the transition zone (TZ) should be clearly labelled as the 2 cell rows at the medial edge of the neuroepithelium (with arrowhead pointing to the cells, not in-between cell types).
4. A schematic depicting timepoints of clonal induction and analysis relative to the initiation timepoint of NE-NB transition, i.e., around 60h ALH would improve the clarity, especially in Figure 2, in addition to the explanation in the materials and methods.
5. Positive results should be moved to the main Figure section, such as the EdU incorporation, and Notch rescue experiments.
6. The authors should double-check their figure call outs. For example, in page 19, Fig S8F’ should have been Fig S9F’.
7. Discussion, page 17: the authors mentioned that Br and Eip93F specify optic lobe neuroblasts based on Fig 7F, H which showed reduced Br and Eip93F expressions upon Snr1 knockdown. This doesn’t seem right. In that same paragraph the authors suggested that “Snr1 contributes to expression of appropriate cell type markers…”. “Cell type markers” is a vague expression, are they spatial and temporal transcription factors?
8. Statistics: the authors used Student’s t-test for the majority of 2 sample comparison. However, much of the data appears not normally distributed, non-

© 2023. Published by The Company of Biologists under the terms of the Creative Commons Attribution License (https://creativecommons.org/licenses/by/4.0/).

parametric tests should be used instead.

Reviewer 2

Advance summary and potential significance to field

The authors greatly improved the manuscript and I recommend publication after addressing the following issues:

Comments for the author

- « Among the mRNAs with the highest relative expression in neuroepithelial cells were Notch target genes ». Please indicate compared to what.

- “Strikingly, we not only found that Snr1R3 mutant clones persisted into the adult brain, but they were Dpn+ (Fig. 4H, H’)”
I suggest to change “they were Dpn+” by “they contained a small subset of Dpn+ cells”
- Drosophila should be written in italics
- In the abstract, the authors write
“Thus, Snr1 regulates the chromatin state in neuroepithelial cells and maintains chromatin state in neural stem cells for proper brain development.”

And in the discussion:

The authors state:

“…Based on our findings, Snr1 is needed to maintain open chromatin at Notch target genes…”
“… We find that Snr1 contributes to establishing a chromatin state defining neuroepithelial cells and maintaining this state in neuroblasts…”
I suggest toning down the claims that Snr1 “regulates the chromatin state”, “maintains the chromatin open” or “establishes a chromatin state”. Indeed, there is no evidence in this manuscript that chromatin accessibility is altered upon Snr1 knockdown, and I don’t think this has been shown in flies. The authors would need to do ATAC-seq experiments in the wt and knockdown context to make these claims.
- Please add a list of differentially expressed genes between control and Snr1RNAi neuroblast, as was done for neuroepithelial cells (Table S1).
- « Clones did not have cells with abnormal perdurance of Dpn expression (Fig. S9E, F), but did have altered morphology, appearing more clustered (Fig S8F’) compared to the columnar organization of the control »
Please change Fig S8F’ by Fig S9F’.

Second revision

Author response to reviewers' comments

We thank the reviewers for their suggestions and corrections. We have addressed all of the remaining comments and again feel that they have further improved our manuscript. Please see our detailed responses to each reviewer below. The reviewer comments are included in italics. We look forward to hearing from you

Reviewer 1 Advance Summary and Potential Significance to Field:
See previous review comments

© 2023. Published by The Company of Biologists under the terms of the Creative Commons Attribution License (https://creativecommons.org/licenses/by/4.0/).

Reviewer 1 Comments for the Author:

-The authors have addressed most of my concerns. There are a few more modifications that can be made in order to improve the manuscript.

1. Regarding the epistatic tests of Snr1 with Br and Eip93F requested, the authors have only done Br and Eip93F knockdown by RNAi, which phenocopied Snr1RNAi. They have not done epistasis of Br or Eip93F and Snr1. I suggest they change the wording where they say they have done the epistasis, and just say that Br and Eip93F knockdown phenocopied Snr1 RNAi.

The description of this on page 11 has been changed as follows: “Given that *Snr1*^{RNAi} reduced levels of *br*, *Eip93F* and *Notch* pathway genes, we tested if reducing expression of each gene would recapitulate the phenotype seen in *Snr1*^{RNAi} clones. Clones expressing *Notch*^{RNAi} or *br*^{RNAi} did resemble *Snr1*^{RNAi} clones with ectopic Dpn+ cells seen deep in the medulla (Fig. S8B, C, D), however *Eip93F*^{RNAi} clones did not (Fig. S8E).”

2. Description of optic lobe development needs to be a bit more comprehensive. In the result 1, the idea of “outer neuroepithelium” appeared quite abruptly, which should be first mentioned in the introduction. Inspirations could be found in the review of Apitz and Salecker, 2014.

A more in depth description of optic lobe development mentioning the outer neuroepithelium has been included in the introduction (page 3).

3. In Figure 1, the transition zone (TZ) should be clearly labelled as the 2 cell rows at the medial edge of the neuroepithelium (with arrowhead pointing to the cells, not in-between cell types).

This has been changed to a bracket in Fig. 1B, 1E-E’’, 2G-H’’’, 6C-D’’.

4. A schematic depicting timepoints of clonal induction and analysis relative to the initiation timepoint of NE- NB transition, i.e., around 60h ALH would improve the clarity, especially in Figure 2, in addition to the explanation in the materials and methods.

A timeline is now included as Fig. 2A. Description of the timing in the materials and methods also refers to Fig. 2A.

5. Positive results should be moved to the main Figure section, such as the EdU incorporation, and Notch rescue experiments.

The results of the EdU incorporation experiment have now been added as Fig. 3F-H as this provides an explanation to the reduced size of the Snr1 clones. The Notch or Notch target gene [E(spl)] rescue experiments showed a partial rescue of the Snr1 phenotype and not all Notch target genes did rescue the Snr1 phenotype. Therefore we kept the Notch rescue experiment as Figure S7 as we think this is required to ensure clarity of the results in the main figures.

6. The authors should double-check their figure call outs. For example, in page 19, Fig S8F’ should have been Fig S9F’.

Thank you. This has been corrected and we have carefully checked the remainder of the text to ensure all call outs are correct.

7. Discussion, page 17: the authors mentioned that Br and Eip93F specify optic lobe neuroblasts based on Fig 7F, H which showed reduced Br and Eip93F expressions upon Snr1 knockdown. This doesn’t seem right. In that same paragraph, the authors suggested that “Snr1 contributes to expression of appropriate cell type markers…”. “Cell type markers” is a vague expression, are they spatial and temporal transcription factors?

This paragraph has been clarified and the term “cell type markers” has been removed (page 17).

© 2023. Published by The Company of Biologists under the terms of the Creative Commons Attribution License (https://creativecommons.org/licenses/by/4.0/).

8. Statistics: the authors used Student's t-test for the majority of 2 sample comparison. However, much of the data appears not normally distributed, non-parametric tests should be used instead.

We appreciate this comment and the statistics have been recalculated using the non-parametric Wilcoxon rank sum test or the Kruskal-Wallis test. These results have been updated in the relevant figures and the materials and methods section.

Reviewer 2 Advance Summary and Potential Significance to Field:
The authors greatly improved the manuscript and I recommend publication after addressing the following issues:

Reviewer 2 Comments for the Author:

- « Among the mRNAs with the highest relative expression in neuroepithelial cells were Notch target genes ». Please indicate compared to what.

This has been clarified on page 11. It now reads "Among the mRNAs with the highest relative expression in this cluster compared to other cell type clusters in the dataset were Notch target genes…".

- "Strikingly, we not only found that Snr1R3 mutant clones persisted into the adult brain, but they were Dpn+ (Fig. 4H, H')" I suggest to change "they were Dpn+" by "they contained a small subset of Dpn+ cells"

This has been changed as suggested on page 9.

- Drosophila should be written in italics

This has been changed as suggested throughout the manuscript.

In the abstract, the authors write "Thus, Snr1 regulates the chromatin state in neuroepithelial cells and maintains chromatin state in neural stem cells for proper brain development."

And in the discussion: The authors state:
"…Based on our findings, Snr1 is needed to maintain open chromatin at Notch target genes…"

"… We find that Snr1 contributes to establishing a chromatin state defining neuroepithelial cells and maintaining this state in neuroblasts…"

I suggest toning down the claims that Snr1 "regulates the chromatin state", "maintains the chromatin open" or "establishes a chromatin state". Indeed, there is no evidence in this manuscript that chromatin accessibility is altered upon Snr1 knockdown, and I don't think this has been shown in flies. The authors would need to do ATAC-seq experiments in the wt and knockdown context to make these claims.

We have toned down our wording to accurately reflect the extent of our results within the context of the reviewer's accurate point. In the abstract we have modified the text to "Snr1 likely regulated the chromatin state in neuroepithelial cells and maintains chromatin state in neural stem cells for proper brain development."(page 2). In the discussion, on page 16 we modified the text to "We propose that Snr1 contributes to establishing a chromatin state defining neuroepithelial cells and maintaining this state in neuroblasts." On page 17 we modified the text to " Based on our finding, Snr1 is likely needed to maintain open chromatin at Notch target genes."

- Please add a list of differentially expressed genes between control and Snr1RNAi neuroblast, as was done for neuroepithelial cells (Table S1).

This has been included as Table S2.

© 2023. Published by The Company of Biologists under the terms of the Creative Commons Attribution License (https://creativecommons.org/licenses/by/4.0/).

- « Clones did not have cells with abnormal perdurance of Dpn expression (Fig. S9E, F), but did have altered morphology, appearing more clustered (FigS8F') compared to the columnar organization of the control » Please change Fig S8F' by Fig S9F'.

This has been corrected.

Third decision letter

MS ID#: DEVELOP/2022/201484

MS TITLE: A chromatin remodelling SWI/SNF subunit Snr1 regulates neural stem cell determination and differentiation

AUTHORS: Sophie E Keegan, Julie Haskins, Andrew J Simmonds, and Sarah C Hughes
ARTICLE TYPE: Research Article

I am delighted to tell you that your manuscript has been accepted for publication in Development, pending our standard ethics checks.

© 2023. Published by The Company of Biologists under the terms of the Creative Commons Attribution License (https://creativecommons.org/licenses/by/4.0/).
